# Superior Sodium Metal Anodes Enabled by 3D Hierarchical Metallic Scaffolds with Enhanced Sodiophilicity

**DOI:** 10.1002/advs.202500756

**Published:** 2025-04-18

**Authors:** Chong Chen, Rui Yang, Jie Zhu, Wenjiao Yao, Yongbing Tang

**Affiliations:** ^1^ Advanced Energy Storage Technology Research Center Shenzhen Institutes of Advanced Technology Chinese Academy of Sciences Shenzhen 518055 China; ^2^ University of Chinese Academy of Sciences Beijing 100049 China; ^3^ Shenzhen Key Laboratory of Energy Materials for Carbon Neutrality Shenzhen 518055 China

**Keywords:** 3D conductive scaffolds, dendrites, hollow nanotube arrays, Na metal anodes, sodiophilicity

## Abstract

Sodium‐metal batteries (SMBs) are regarded as key for next‐generation energy storage due to their high theoretical energy and potential cost effectiveness. However, Na‐metal systems remain challenging by critical barriers, including severe Na dendrites growth and infinite huge volume change, which limit the feasibility of SMBs. Here, this work develops a 3D conductive scaffold consisting of vertical crystalline TiO_2_ nanotube arrays embedded with ultrafine silver nanoparticles (denoted as Ag@TiO_2_ NTAs) with ultrasonication‐assisted in situ deposition method for high‐performance SMBs. Significantly, the hierarchical hollow nanotubes with large surface area can reduce the current density to promote compact electrodeposition and guide the parallel growth of Na. Meanwhile, the sodiophilic Ag nanocrystals with strong interactions with Na^+^ enable a marked reduction of the nucleation barriers. As a result, the Na metal anode with the Ag@TiO_2_ NTAs host delivers remarkable electrochemical properties including ultralow voltage hysteresis and prolonged cycling stability over 3600 h. By pairing with a Na_3_V_2_(PO_4_)_3_ cathode, the SMBs achieve 87% capacity retention after 2000 cycles at 8 C, suggesting its potential application for highly stable Na anodes.

## Graphical abstract

This work develops a 3D conductive scaffold consisting of vertical crystalline TiO_2_ nanotube arrays embedded with ultrafine silver nanoparticles (denoted as Ag@TiO_2_ NTAs) with an ultrasonication‐assisted in situ deposition method for high‐performance Na metal batteries. Significantly, the hierarchical hollow nanotubes reduce the current density and guide the parallel growth of Na. Meanwhile, the sodiophilic Ag nanocrystals enable a marked reduction of the nucleation barriers. As a result, the Na metal anodes with the Ag@TiO_2_ NTAs host deliver remarkable electrochemical properties.

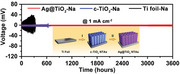

## 1 Introduction

The rapid development of industry and the increasing wealth of electricity‐based society highlights the great importance of a consistent energy supply.^[^ ^1^ ^]^ Current lithium‐ion batteries (LIBs) based on the reversible insertion/extraction of Li^+^ in the oxide cathodes (layered, spinel, polyanion families, etc.) and graphite anodes with liquid electrolyte reach a specific energy of ∼260 Wh·kg^−1^ and energy density of ∼770 Wh·L^−1^ and will soon approach their theoretical limit.^[^ ^2^ ^]^ Nonetheless, the concerns regarding long‐term Li precursor fluctuations and the material availability are still very challenging.^[^ ^3^ ^]^ Sodium‐metal batteries (SMBs) have received intensive scientific attention to replace the state‐of‐the‐art LIBs owing to their superiorities such as environmental friendliness, cost advantage, high theoretical specific capacity (1166 mAh g^−1^), and low standard electrode potential (−2.71 V versus standard hydrogen electrode, SHE).^[^ ^4^ ^]^ However, Na‐metal systems still face great obstructions, stemming from the un‐disciplinable dendrites with most organic electrolytes and infinite volume change during sodiation‐desodiation processes, which gives rise to poor battery cycling stability and premature cell failure.^[^ ^5^ ^]^

To practically tackle the above issues, several complementary strategies have been employed to stabilize Na metal anodes and prevent dendrite formation with varying success.^[^ ^6^ ^]^ Of note, the utilization of host structures has received attention in the literature, having the key advantage of being readily directly integrated into the current collector, or being the current collector itself.^[^ ^7^ ^]^ Unoccupied pore space in the host may shield the Na metal from excessive reactions and buffer a portion of the volume changes for the plating Na metal.^[^ ^8^ ^]^ Compared with planar current collector, the host systems can impede concentration polarization driven dendrite growth, since the metal is now being plated onto 3D scaffolds that may be up to several orders of magnitude higher surface area than the underlying planar configurations.^[^ ^9^ ^]^ A variety of porous materials have been employed as hosts for Na metal anodes, among which, porous metals (Al, Zn, Cu, and Ni) with good electrical conductivity have been proven to be promising candidates.^[^ ^10^ ^]^ Unfortunately, Na metal can hardly be evenly distributed and guided inside the scaffold during the plating process, thereby inducing inhomogeneous Na deposition.^[^ ^11^ ^]^ Instead, Na metal tends to aggregate and deposit at the top surface of the scaffold, thus resulting in the dendritic Na‐overgrowth issue.^[^ ^12^ ^]^ Over time, the accumulated Na on the exterior may further blocks inward ion‐transport pathways accessing to the internal scaffolds, which increases electrochemical instability at the anode.^[^ ^13^ ^]^ Therefore, manipulating the micro‐nano structure of hosts with sodiophilic surfaces and guiding the parallel growth of Na are important for stabilizing Na metal anodes.

Here, we report the delicate design and synthesis of 3D crystalline TiO_2_ nanotube arrays (c‐TiO_2_ NTAs) decorated with ultrafine silver nanocrystals (denoted as Ag@TiO_2_ NTAs) as a composite host for Na metal anodes. Compared with bare Ti foil and c‐TiO_2_ NTAs without Ag nanoparticle decoration, the Ag@TiO_2_ NTAs hosts exhibit apparent advantages. First, the 3D conductive framework with a large surface area reduces the current density to promote relatively compact electrodeposition and restrict the dimension change. Meanwhile, the well‐designed hierarchical vertical nanotubes provide enough space for Na deposition and enhance structural stability by buffering volume changes. The sodiophilic Ag nanoparticles with strong Na^+^ interactions ensure uniform Na deposition on the interior and exterior surfaces of nanotubes by refining nucleation barriers.^[^ ^14^ ^]^ Benefiting from these superiorities, the Ag@TiO_2_ NTAs electrode shows decreased nucleation overpotential for dendrite‐free Na deposition with a low voltage hysteresis and superior cycling stability over 3600 h. Furthermore, the Na metal full cells coupled with the Na metal anode confined in the Ag@TiO_2_ NTAs host and Na_3_V_2_(PO_4_)_3_ (NVP) cathode deliver outstanding cycling stability and rate capability, suggesting its great potential for practical applications.

## 2 Results and Discussion

**Figure 1:**
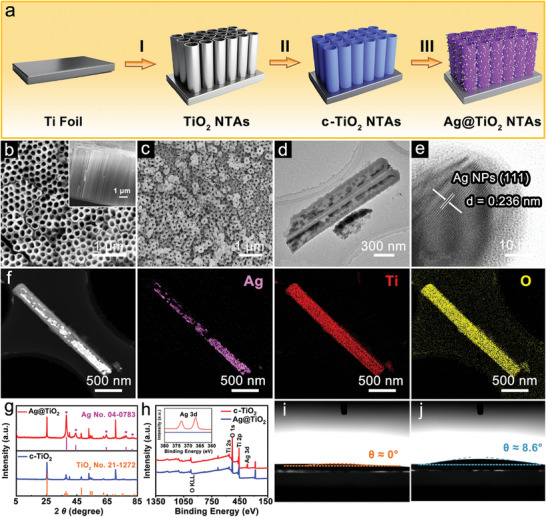
a) Schematic illustration of the synthetic process for Ag@TiO_2_ NTAs hosts. b) FESEM images of c‐TiO_2_ NTAs. The inset shows the cross‐sectional image of c‐TiO_2_ NTAs. c) FESEM, d) TEM and e) HRTEM images of Ag@TiO_2_ NTAs. f) HAADF‐STEM and the corresponding elemental mapping images of a single Ag@TiO_2_ nanotube. g) XRD patterns of Ag@TiO_2_ and c‐TiO_2_ NTAs. h) XPS survey spectra of Ag@TiO_2_ and c‐TiO_2_ NTAs. Contact angle tests of diglyme electrolyte on i) Ag@TiO_2_ NTAs and j) bare Ti foils.

The overall synthesis route of Ag@TiO_2_ NTAs is schematically illustrated in **Figure** **1**a. Typically, TiO_2_ NTAs precursors are prepared by the classical electrochemical anodic oxidation method, and thus obtaining the crystalline c‐TiO_2_ NTAs through calcination at 400 °C.^[^ ^15^ ^]^ Ag@TiO_2_ NTAs are synthesized by dispersing silver nanocrystals on vertically aligned c‐TiO_2_ NTAs with an ultrasonication‐assisted in situ deposition method.^[^ ^16^ ^]^ Under ultrasonication, Ag^+^ ions diffuse and penetrate into the nanotubes. After adding glucose into the reaction system, Ag^+^ ions are reduced to Ag particles and grow on and inside the c‐TiO_2_ nanotubes. The digital photographs show the color of the corresponding products change from shiny luster to black due to the loading of Ag nanocrystalline (Figure S1, Supporting Information).

Field‐emission scanning electron microscopy (FESEM) images reveal the dense and mechanically strong structure of the Ti foil (Figure S2, Supporting Information). After the electrochemical anodic oxidation process, uniform and highly oriented TiO_2_ NTAs are successfully grown on the Ti substrate (Figure S3a,b, Supporting Information). The diameter and average length of the TiO_2_ nanotubes are estimated to be 160 nm and 8 µm, respectively. X‐ray diffraction (XRD) pattern shows that the TiO_2_ NTAs is in amorphous phase (Figure S3c, Supporting Information). In situ XRD measurements reveal the successive structural transformations from pristine amorphous to anatase then subsequently to a rutile phase during heating from room temperature to 800 °C (Figure S4, Supporting Information). To increase the structural stability, TiO_2_ NTAs are calcined at 400 °C in air conditions to obtain the crystalline c‐TiO_2_ NTAs. The tubular morphology of the materials is well maintained (Figure 1b; Figure S5a,b, Supporting Information). Meanwhile, the transmission electron microscopy (TEM) observation reveals a tubular configuration with a smooth surface (Figure S5c, Supporting Information). The high‐angle annular dark‐field scanning TEM (HAADF‐STEM) image and the corresponding energy‐dispersive X‐ray (EDX) spectroscopy elemental mapping images reveal the uniform distribution of Ti and O species throughout the hollow nanotubes (Figure S5d–f, Supporting Information). To provide a sodiophilic interface and a stable matrix to accommodate Na metal, ultrathin Ag nanoparticles are then decorated on the edge and inner wall of c‐TiO_2_ NTAs. It should be mentioned that Ag@TiO_2_ NTAs inherit the structural features from the c‐TiO_2_ NTAs precursors without apparent external changes (Figure 1c; Figure S6a, Supporting Information). TEM observation elucidates the presence of the Ag nanocrystals (Figure 1d; Figure S6b, Supporting Information). Apart from the nanotubes, homogeneous Ag nanoparticles can be observed on the agglomerating particles attached to the inner shells. Fewer Ag nanocrystals grow at the outer wall of the nanotube due to the limited space. The 1D nanotube channel is still in reserve. The high‐resolution TEM (HRTEM) image shows the lattice fringe with a spacing distance of 0.236 nm, which corresponds to the (111) plane of Ag (Figure 1e).^[^ ^17^ ^]^ The HAADF‐STEM and corresponding elemental mapping images depict the homogeneous distribution of the Ag nanoparticles throughout atop the surface of c‐TiO_2_ nanotubes (Figure 1f; Figure S6c–f, Supporting Information). The cross‐sectional FESEM image shows rough surfaces with coarse Ag agglomerates (Figure S7, Supporting Information). The EDX results at cross‐section view also verify the existence of Ag (Figure S8, Supporting Information). XRD patterns further reveal the successful fabrication of Ag@TiO_2_ NTAs. As shown in Figure 1g, all of the characteristic peaks are well in accordance with the metallic Ag phase [Joint Committee on Powder Diffraction Standards (JCPDS) No. 04‐0783], indicating that the ultrathin nanoparticles anchored in c‐TiO_2_ NTAs are Ag nanocrystals.^[^ ^18^ ^]^ X‐ray photoelectron spectroscopy (XPS) measurement is used to analyze the chemical composition and valence state for Ag@TiO_2_ and c‐TiO_2_ NTAs (Figure 1h; Figure S9, Supporting Information).^[^ ^19^ ^]^ XPS spectra prove the existence of Ag, Ti, and O elements in the Ag@TiO_2_ NTAs composite. To evaluate the Na ion migration kinetic for the Ag@TiO_2_ NTAs, contact angles of diglyme electrolyte are measured as shown in Figure 1i,j and Figure S10 (Supporting Information).^[^ ^20^ ^]^ The contact angle of electrolyte on the surface of Ag@TiO_2_ NTAs is ≈0°, which is smaller than that of Ti foil (8.6°) and c‐TiO_2_ NTAs (6.8°). The notably decreased contact angle reflects the strong capillary actions on the Ag@TiO_2_ NTAs surface, which is beneficial to the lateral growth of Na crystals.

**Figure 2:**
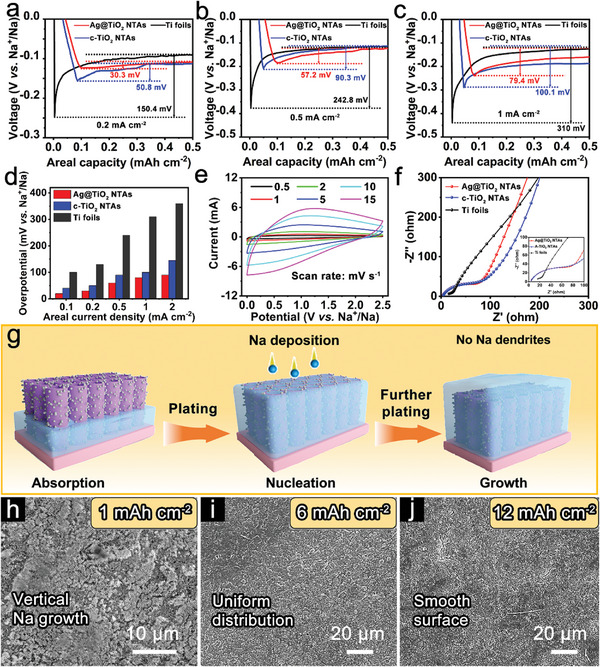
Nucleation overpotential profile of Na plating on different hosts at a) 0.2 mA cm^−2^, b) 0.5 mA cm^−2^ and c) 1 mA cm^−2^. d) Summary of the Na plating overpotentials on the various hosts. e) CV curves of Ag@TiO_2_ NTAs in half cells. f) EIS Nyquist plots of different substrates. g) Schematic illustration of Na deposition on the Ag@TiO_2_ NTAs. FEESM images of the Ag@TiO_2_ NTAs hosts after Na plating with a capacity of h) 1 mAh cm^−2^, i) 6 mAh cm^−2^ and j) 12 mAh cm^−2^.

To evaluate the effectiveness of the multifunctional component for the nucleation and growth during the Na deposition, the electrochemical performances of Ag@TiO_2_ NTAs, c‐TiO_2_ NTAs and bare Ti foils are investigated. The Coulombic efficiencies (CEs) for the Na metal anodes with different hosts are presented in Figure S11 (Supporting Information). Before the CE test, the cells were discharged and charged at a low current density of 0.1 mA cm^−2^ for pre‐SEI formation. The CE of Ag@TiO_2_ NTAs shows a quite stable plating/ stripping process of 99.3% for 200 cycles at areal capacity of 3 mAh cm^−2^ with 1 mA cm^−2^. In contrast, the CEs of c‐TiO_2_ NTAs and bare Ti foils provide a rather limited stability. This is ascribed to the large nucleation barrier for Na on the surface of c‐TiO_2_ NTAs and bare Ti, resulting in uneven Na nucleation, and eventually leading to the formation of Na dendrites. The nucleation overpotential for the Na metal plating, which is defined as the voltage gap between the dip and the stable plateau on the voltage curve during the first plating process, is investigated for the host materials at various current densities.^[^ ^21^ ^]^ Notably, the nucleation overpotentials with the Ag@TiO_2_ NTAs hosts at 0.1, 0.2, and 0.5 mA cm^−2^ are 30.3, 57.2, and 79.4 mV, respectively, which are smaller than those for the Na plating on the c‐TiO_2_ NTAs hosts (**Figure** **2**a–c). In comparison, the nucleation overpotentials for the Na plating on the bare Ti foil electrodes at 0.1, 0.2, and 0.5 mA cm^−2^ are 150.4, 242.8, and 310.0 mV, respectively. The large nucleation overpotentials for the planar Ti foil electrodes further demonstrate the advantage of the 3D conducting scaffold with a high‐specific area, which could delay the emergence of Na dendrites by dissipating the effective current density.^[^ ^22^ ^]^ The Ag@TiO_2_ NTAs exhibit the lowest nucleation overpotentials among samples, further showing its superiorities as Na hosts (Figure 2d; Figure S12, Supporting Information). Further, the voltage‐capacity curves at different cycles of the cells are summarized in Figure S13 (Supporting Information). It can be seen that the cycling performance of the Ag@TiO_2_ NTAs half‐cell from the beginning to the 200^th^ cycle is quite stable, resulting in almost overlapping voltage‐capacity curves except for the initial cycle. Cyclic voltammetry (CV) curves prove the ultrafast sodiation kinetics of Ag@TiO_2_ NTAs (Figure 2e).^[^ ^23^ ^]^ All the CV curves exhibit similar shapes and well‐preserved broad redox peaks with the peak currents becoming progressively enlarged along with stepwise increases of the sweep rate. The surface capacitive contribution reaches 87.1% at 15 mV s^−1^, which indicates favorable Na^+^ storage kinetics (Figure S14, Supporting Information). Meanwhile, electrochemical impedance spectroscopy (EIS) spectra show that the Ag@TiO_2_ NTAs exhibit lower charge‐transfer resistance for the promoted ion diffusion kinetics (Figure 2f).^[^ ^24^ ^]^ It is noteworthy that the decreased interfacial resistance for the bare Ti foil can be attributed to the less resistive solid electrolyte interphase (SEI) layer formed with low surface area.^[^ ^25^ ^]^ By fitting the EIS profiles to the equivalent circuit model established at different frequencies, we were able to extract the charge transfer resistance (*R*_ct_) and the cell internal resistance (*R*_SEI_) values corresponding to the two processes, respectively (Figure S15, Supporting Information). The fitted *R*_SEI_ and *R*_ct_ values are shown in Table S1 of the Supporting Information. After the Ag nanoparticles deposition, both *R*_SEI_ and *R*_ct_ are smaller, suggesting that cells with the Ag@TiO_2_ NTAs substrate own better electron/ionic conductivity and charge transfer dynamics. Based on the aforementioned results, Ag@TiO_2_ NTAs display great potential as dendrite‐free SMB hosts (Figure 2g). Owing to the high sodiophilicity and small overpotential for nucleation/growth, Ag@TiO_2_ NTAs enables the uniform Na deposition on both the interior and exterior surfaces without the generation of Na dendrites. Besides, the hierarchical 3D hollow construction provides sufficient space to store metallic Na and effectively suppresses volume expansion during repeated cycling.^[^ ^26^ ^]^ The morphology evolution of the Na metal anodes on bare Ti foils, c‐TiO_2_ NTAs, and Ag@TiO_2_ NTAs is also investigated by top‐view FESEM images at different areal capacities.^[^ ^27^ ^]^ At the plating capacity of 1 mAh cm^−2^, the Ag@TiO_2_ NTAs electrode shows its original 3D framework as well as the nanotubes (Figure 2h). As the areal capacity increases to 6 mAh cm^−2^, the Ag@TiO_2_ NTAs hosts are fully covered by deposited Na metal, and a uniform and compact Na deposition layer can be observed on the surface of Ag@TiO_2_ NTAs (Figure 2i). When the Na plating capacity is continuously increased to 12 mAh cm^−2^ or even at 20 mAh cm^−2^, the Ag@TiO_2_ NTAs electrode can constantly keep a relatively dense and flat Na deposition (Figure 2j; Figure S16, Supporting Information).^[^ ^28^ ^]^ No dendrite‐like morphologies and mossy Na can be detected during the whole Na plating process. EDX mapping further proves the homogeneous Na deposition inside the Ag@TiO_2_ NTAs hosts, which indicates an oriented plating behavior of Na metal along the surface of nanotubes (Figure S17, Supporting Information). In contrast, the c‐TiO_2_ NTAs and bare Ti foils exhibit an irregular surface with many Na nodules (Figure S18, Supporting Information). The surface for the c‐TiO_2_ NTAs is rough and uneven. The Na metal on the bare Ti foils shows much Na lumps and even mossy Na, which is most likely derived from the cracking of large Na nodules, suggesting the poor cycling stability of the Na metal on bare Ti foils.^[^ ^29^ ^]^ The above results demonstrate that the 3D conductive matrix with sodiophilic Ag nanocrystals can guide more uniform Na nucleation and deposition, thereby achieving the effect of suppressing dendrites.

**Figure 3:**
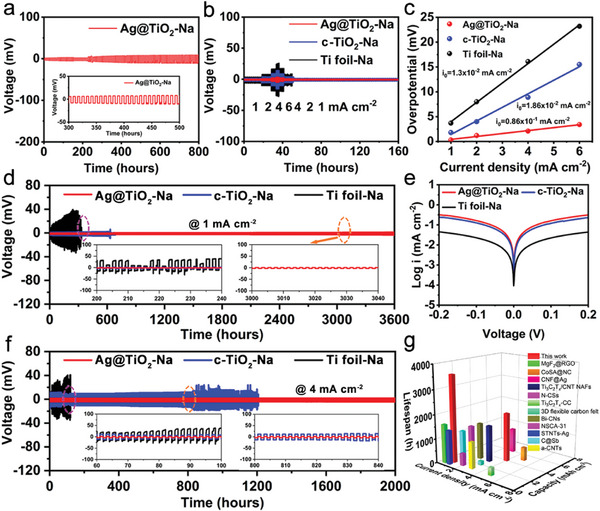
Electrochemical performance of symmetric cells based on different electrodes. a) Galvanostatic discharge and charge voltage profiles using the Ag@TiO_2_‐Na electrodes at 1 mA cm^−2^ for 4 mAh cm^−2^. b) Rate performances of the symmetric cells with Ag@TiO_2_‐Na, c‐TiO_2_‐Na, and Ti foil‐Na from 1 to 6 mA cm^−2^. c) The calculated *i_0_* of Ag@TiO_2_‐Na, c‐TiO_2_‐Na, and Ti foil‐Na. d) Cycling performances of the symmetric Na cells at 1 mA cm^−2^ for 1 mAh cm^−2^. e) Tafel curves of different electrodes. f) Cycling performances of the symmetric Na cells at 4 mA cm^−2^ for 4 mAh cm^−2^. g) Comparison of recently reported hosts for SMBs in symmetric cells.

The plating and stripping performances of these electrodes are further evaluated using symmetrical cells.^[^ ^30^ ^]^ Before symmetrical cell assembly, 6 mAh cm^−2^ of Na is plated onto the Ag@TiO_2_ NTAs, c‐TiO_2_ NTAs, and planar Ti foils at a current density of 1 mA cm^−2^ to form the Ag@TiO_2_‐Na, c‐TiO_2_‐Na, and Ti‐Na. As shown in **Figure** **3**a, the symmetrical cell with Ag@TiO_2_‐Na electrode exhibits a small overpotential (8.5 mV) and achieves stable cycling over 800 h at 1 mA cm^−2^ for 4 mAh cm^−2^. The corresponding detailed voltage profiles of Ag@TiO_2_‐Na electrodes show virtually flat voltage plateaus throughout the cycling. The rate capabilities of the Ag@TiO_2_‐Na, c‐TiO_2_‐Na, and Ti‐Na are conducted by cycling the symmetric cell at various current densities ranging from 1 to 6 mA cm^−2^ with a fixed Na deposition capacity of 1 mAh cm^−2^ (Figure 3b). It can be seen that the Ti foil electrodes exhibit poor stability and the voltage polarization enlarges greatly as the current density increases, which is much higher than those for the Ag@TiO_2_‐Na and c‐TiO_2_‐Na electrodes. This indicates that the 3D conductive structures could effectively decrease the local current density during Na plating/stripping at high current rates, to deliver smaller voltage hysteresis as compared to the 2D planar Ti foils. The Ag@TiO_2_‐Na and c‐TiO_2_‐Na electrodes show better cycling efficiency and they deliver stable cycling for 160 h. It is noticeable that the voltage hysteresis for the Ag@TiO_2_‐Na electrode is relatively smaller than that for the c‐TiO_2_‐Na electrode, which suggests the decoration of Ag nanoparticles on 3D conducting scaffold can regulate Na plating/stripping behavior. The exchange current density (*i_0_*) is calculated to evaluate the Na^+^ diffusion dynamics during Na platting/stripping process based on the Butler‐Volmer approximation equation: $i=i_{0}\frac{\eta F}{2RT}$, where *i* and *η* are the applied current density and overpotential corresponding to the rate test, *F* is the Faraday constant, *R* is the ideal gas constant, and *T* is the Kelvin temperature. The Ag@TiO_2_‐Na electrode delivers a sharply larger value of *i_0_* than that of c‐TiO_2_‐Na and Ti foil, suggesting the accelerated kinetically ions diffusion (Figure 3c). Long‐term cycling for different electrodes is also investigated in symmetric cells (Figure 3d). At a current density of 1 mA cm^−2^ for 1 mAh cm^−2^, the Ag@TiO_2_‐Na symmetric cell exhibits the lowest overpotential with remarkable cycling stability for 3600 h. However, the c‐TiO_2_‐Na and Ti foils‐Na electrodes experience random voltage fluctuations and fast cell failures after cycling of about 270 and 730 h, which might be attributed to the short circuit or detachment of debris Na.^[^ ^31^ ^]^ Tafel curves in Figure 3e are further proofs of the accelerated kinetics and the lower energy barrier enabled by the effective host structure and the sodiophilic functional group on the Ag@TiO_2_ NTAs. Even when further increasing the current density to higher values, the Ag@TiO_2_‐Na anodes could still maintain stable cycling for 2000 h at 4 mA cm^−2^ for 4 mAh cm^−2^ (Figure 3f). The partially enlarged stripping/plating curve of Ag@TiO_2_‐Na is extremely flat without any polarization from the beginning, and the curves approaching to rectangle maintain well after cycled for a long time. These results confirm that the Ag@TiO_2_ NTAs hosts can effectively improve the reversibility of Na plating/stripping. The electrochemical performance of Ag@TiO_2_‐Na is outstanding compared to those of previous studies using different hosts (Figure 3g; Table S2, Supporting Information).

**Figure 4:**
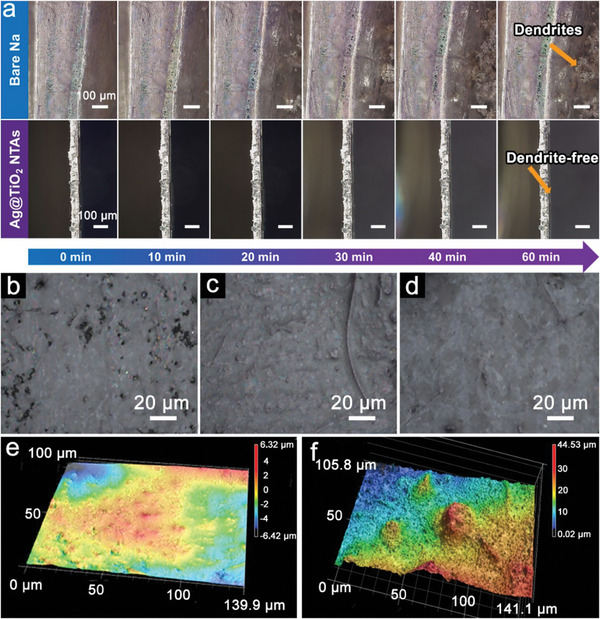
In situ optical microscopy observation of the Na plating process on bare Na metal and the Ag@TiO_2_ NTAs host for different times at a current density of 1 mA cm^−2^. Surface FESEM images of Ti foils electrodes in symmetric cell at 1 mA cm^−2^ after b) 10 cycles, b) 100 cycles, and d) 200 cycles. 3D LSCM images of e) Ag@TiO_2_ NTAs and f) bare Ti foils after 100 cycles at 1 mA cm^−2^.

Considering that the Na deposition is time‐dependent, the in situ optical observation is employed to provide more direct evidence to reveal the Na growth mechanisms. As shown in **Figure** **4**a, the bare Na electrodes exhibit obvious Na dendrite growth with the increase of the plating time at 1 mA cm^−2^ after 20 min. Some small protrusions appear on the surface of Na foil at the early stage and rapidly evolve into mossy‐like Na dendrites with dramatic fluctuation. In contrast, the Ag@TiO_2_ NTAs show negligible changes with a smooth surface during the same plating process, indicating the excellent regulation function in guiding the non‐uniform Na^+^ flux. FESEM analysis of cycled Na metal anodes with the Ag@TiO_2_ NTAs hosts also indicates good sodiophilicity of the conductive scaffold with dense and compact morphology (Figure 4b–d). After 200 cycles, the Na metal plating on the Ag@TiO_2_ NTAs exhibits a uniform and flat interface, while the surface for the c‐TiO_2_ NTAs is rough and uneven (Figure S19, Supporting Information). Similarly, many nodules can be seen in the case of the Na metal plating on the bare Ti foil (Figure S20, Supporting Information), while there are also some Na whiskers, which is most likely derived from the cracking of large Na nodules, suggesting the poor cycling stability of the Na metal on bare Ti foil. Furthermore, the surface morphology evolution of Na deposited on Ag@TiO_2_ NTAs, c‐TiO_2_ NTAs and bare Ti foil substrates are investigated by laser scanning confocal microscopy (LSCM) after 100 cycles at a current density of 1.0 mA cm^−2^ and a capacity of 1.0 mAh cm^−2^ (Figure 4e,f; Figure S21, Supporting Information). As per the 3D surface profilometry images, there are minor variations in local height observed on the cycled Ag@TiO_2_‐Na electrode, while c‐TiO_2_‐Na and Ti foil‐Na exhibit rough and uneven surfaces with a great height difference. From this, it can be deduced that the Ag@TiO_2_ NTAs could significantly enhance the cycling stability of the Na metal anodes, as a result of the 3D confining host and Ag‐derived stable interfaces.

**Figure 5:**
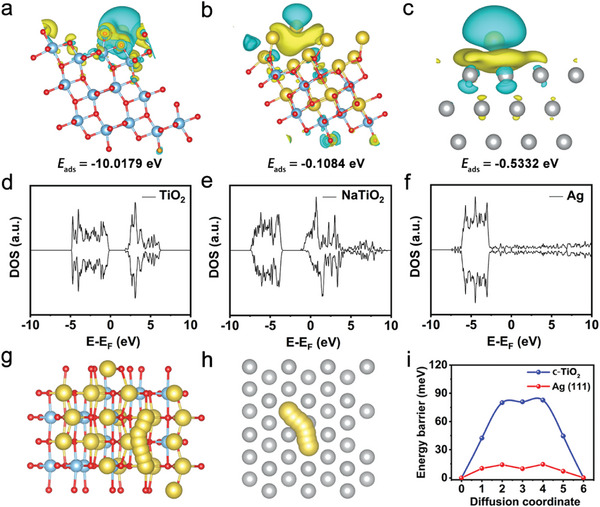
DFT calculations. Charge density differences of the a) c‐TiO_2_, b) NaTiO_2_ and c) Ag (111). The yellow region represents charge accumulation and the cyan region represents charge depletion. Density of states of d) c‐TiO_2_, e) NaTiO_2_ and f) Ag (111). Scheme of Na^+^ migration path inside the g) c‐TiO_2_ and h) Ag (111). The gray, yellow, blue and red spheres indicate Ag, Na, Ti, and O atoms, respectively. i) Calculated energy barrier of Na^+^ diffusion.

Density functional theory (DFT) calculations are performed to further study the Na deposition properties. The adsorption behavior of Na atom on the surface of c‐TiO_2_, fully sodiated c‐TiO_2_, and Ag are first simulated. **Figure** **5**a‐c illustrate the most stable Na adsorption sites on each surface, the corresponding adsorption energy, and the changes in charge density after adsorption of Na atoms. Because the c‐TiO_2_ surface is enriched with a large number of defects, the c‐TiO_2_ surface demonstrates an extremely high sodiophilicity at the initial stage of deposition, with adsorption energy up to −10.02 eV. As the Na storage sites and defects of c‐TiO_2_ surface are saturated with adsorbed Na atoms, the sodiophilicity of c‐TiO_2_ surface diminished, and the adsorption energy of Na atoms are reduced to −0.11 eV. Whereas the adsorption energy of Na atom on Ag (111) surface is −0.53 eV, showing a more sodiophilic property. Consequently, loading Ag nanoparticles on the surface of c‐TiO_2_ nanotubes enhances the affinity of c‐TiO2 substrate for Na, and thereby promotes uniform Na nucleation. Figure 5d–f shows the density of states (DOSs) of c‐TiO_2_, fully sodiated c‐TiO_2_ and Ag. c‐TiO_2_ exhibits a wide band gap, suggesting its poor conductivity. Although the conductivity of c‐TiO_2_ is significantly enhanced after Na doping, the conductivity of semiconductive NaTiO_2_ is inferior to metallic Ag with continuous bands. Therefore, loading Ag nanoparticles on c‐TiO_2_ nanotubes also enhances the conductivity of Na deposition substrate. Moreover, the energy barriers of Na migration on Ag (111) facet and fully sodiated c‐TiO_2_ are further calculated. The most possible migration paths are illustrated in Figure 5g,h. Apparently, the migration energy barrier of Na atom on the Ag (111) facet is remarkably lower than that on the fully sodiated c‐TiO_2_. The Ag nanoparticles facilitate the rapid migration of deposited Na to achieve a uniform distribution on the substrate. According to theoretical calculations, Ag nanoparticles improve Na deposition by enhancing the sodiophilicity and electric conductivity of the substrate, and lowering the Na migration energy barriers.

**Figure 6:**
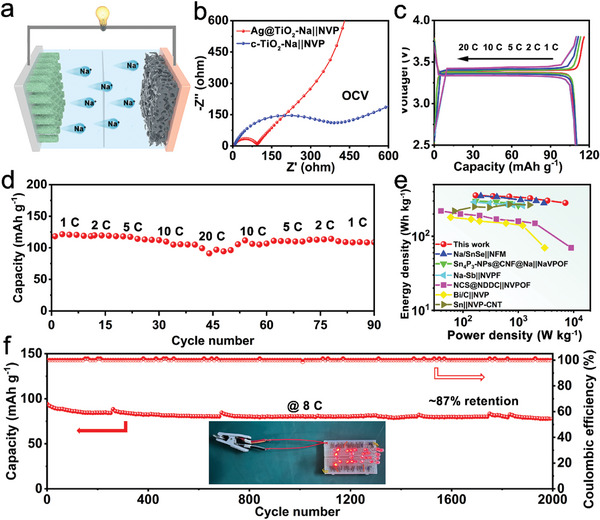
a) Schematic illustration of the Ag@TiO_2_‐Na||NVP full cell. b) Nyquist plots of full cells with different electrodes. c) Discharge/charge curves and d) rate capabilities of Ag@TiO_2_‐Na||NVP full cells at different current rates from 1 to 20 C (1C = 118 mA g^−1^). e) Comparison of energy density in this work and previously reported research in SMBs. f) Cycling stability of Ag@TiO_2_‐Na||NVP full cell at 8 C. Inset: the optical photograph showing that red LED arrays are lightened by Ag@TiO_2_‐Na||NVP device.

To further verify the feasibility of the Ag@TiO_2_ NTAs hosts for practical Na metal anodes, Na metal full cells with a Na_3_V_2_(PO_4_)_3_ (NVP) cathode (Figure S22, Supporting Information) are investigated (**Figure** **6**a). EIS spectra show that the Ag@TiO_2_‐Na||NVP cell exhibits lower charge‐transfer resistance for the promoted ion diffusion kinetics (Figure 6b). The discharge and charge curves of the Ag@TiO_2_‐Na||NVP full cell at different current rates exhibit similar profiles with smaller polarization than those of the bare Na metal full cell (Figures S23 and S24, Supporting Information), confirming the enhanced reaction dynamics and high reversibility of the electrochemical reactions (Figure 6c). The rate performance of the Ag@TiO_2_‐Na||NVP cell is presented in Figure 6d. At a low current density of 1 C, the Ag@TiO_2_‐Na||NVP cell exhibits an average discharge capacity of 121.4 mAh g^−1^. With the increase in current density, the Ag@TiO_2_‐Na||NVP cell still delivers superior discharge capacities of 119.6, 117.3, 110.0, and 99.3 mAh g^−1^ at the current densities of 2, 5, 10, and 20 C, respectively. The Ag@TiO_2_‐Na||NVP cell shows much better rate performance than that of previously reported SMBs (Figure 6e; Table S3, Supporting Information).^[^ ^32^ ^]^ Moreover, the prepared Ag@TiO_2_‐Na||NVP cell also exhibits remarkable cycling stability. The Ag@TiO_2_‐Na||NVP cell can be stably cycled for over 200 cycles at 1 C with 98% capacity retention and an average CE of 99.9% (Figure S25, Supporting Information). Even with a limited amount of Na (1 mAh cm^−2^), the Ag@TiO_2_‐Na||NVP with a low area capacity ratio of negative to positive electrodes (N/P ratio ≈4.5) could still deliver stable cycling for more than 150 cycles (Figure S26, Supporting Information). Also, the cell could be operated steadily for more than 2000 cycles with a high capacity retention of 87% at a large current density of 8 C (Figure 6f). Meanwhile, the Coulombic efficiency reaches almost 100%, demonstrating a highly reversible Na plating‐stripping process on the Ag@TiO_2_‐Na anode. These results further confirm the superiority of the Ag@TiO_2_‐Na as the composite Na anode. In addition, the practical application of the Ag@TiO_2_‐Na||NVP device are tested by lightening up a light‐emitting diode (LED) system. As seen in the inset of Figure 6f and Figure S27 (Supporting Information), the Ag@TiO_2_‐Na||NVP device are able to power and lighten up a SIAT logo consisting of 27 LEDs in parallel, demonstrating its high‐power characteristic and the practical application potential.

## 3 Conclusions

In summary, we develop a 3D crystalline TiO_2_ nanotube arrays decorated with sodiophilic silver nanoparticles as a composite host for SMBs. The resultant 3D framework can provide sufficient surface area for Na nucleation and improve Na plating‐stripping homogeneity, whereas the high sodiophilicity of the Ag nanocrystals strengthens the adhesion and conduction ability, and guarantees the high stability of the nanostructure. Therefore, the symmetric cell with Ag@TiO_2_‐Na electrodes displays superior Na plating‐stripping stability over 3600 h at 1 mA cm^−2^ with a low overpotential. Furthermore, the Ag@TiO_2_‐Na||NVP full cell implements outstanding cycle stability, retaining 87% capacity at 2000 cycles at a high current density of 8 C. This work offers new inspiration for design and synthesis of high‐performance Na anodes toward efficient energy conversion and storage.

## Conflict of Interest

There are no conflicts to declare.

## Supporting information



Supporting Information

## Data Availability

The data that support the findings of this study are available from the corresponding author upon reasonable request.

## References

[1] a) H. C. Hao , T. Hutter , B. L. Boyce , J. Watt , P. C. Liu , D. Mitlin , Chem. Rev. 2022, 122, 8053; 35349271 10.1021/acs.chemrev.1c00838

[2] a) J. Holoubek , K. Kim , Y. Yin , Z. Wu , H. Liu , M. Li , A. Chen , H. Gao , G. Cai , T. A. Pascal , P. Liu , Z. Chen , Energy Environ. Sci. 2022, 15, 1647;

[3] a) L. M. Morgan , M. M. Islam , H. Yang , K. O'Regan , A. N. Patel , A. Ghosh , E. Kendrick , M. Marinescu , G. J. Offer , B. J. Morgan , M. S. Islam , J. Edge , A. Walsh , ACS Energy Lett. 2022, 7, 108;

[4] a) J. Pu , C. L. Zhong , J. H. Liu , Z. H. Wang , D. L. Chao , Energy Environ. Sci. 2021, 14, 3872;

[5] a) X.‐Y. Cui , Y.‐J. Wang , H.‐D. Wu , X.‐D. Lin , S. Tang , P. Xu , H.‐G. Liao , M.‐S. Zheng , Q.‐F. Dong , Adv. Sci. 2021, 8, 2003178; 10.1002/advs.202003178 PMC7816717 33511020

[6] a) C. Zhang , A. Wang , J. Zhang , X. Guan , W. Tang , J. Luo , Adv. Energy Mater. 2018, 8, 1802833;

[7] a) K. Lee , Y. J. Lee , M. J. Lee , J. Han , J. Lim , K. Ryu , H. Yoon , B.‐H. Kim , B. J. Kim , S. W. Lee , Adv. Mater. 2022, 34, 2109767; 10.1002/adma.202109767 35133699

[8] a) H. Wang , W. Bai , H. Wang , D. Kong , T. Xu , Z. Zhang , J. Zang , X. Wang , S. Zhang , Y. Tian , X. Li , C.‐S. Lee , Y. Wang , Energy Storage Mater. 2023, 55, 631;

[9] a) X. Lu , H. Zhao , Y. Qin , E. Matios , J. Luo , R. Chen , H. Nan , B. Wen , Y. Zhang , Y. Li , Q. He , X. Deng , J. Lin , K. Zhang , H. Wang , K. Xi , Y. Su , X. Hu , S. Ding , W. Li , ACS Nano 2023, 17, 10665; 37227175 10.1021/acsnano.3c01759

[10] a) Y. Xu , A. S. Menon , P. P. R. M. L. Harks , D. C. Hermes , L. A. Haverkate , S. Unnikrishnan , F. M. Mulder , Energy Storage Mater. 2018, 12, 69;

[11] a) D. Luo , M. Li , Y. Zheng , Q. Ma , R. Gao , Z. Zhang , H. Dou , G. Wen , L. Shui , A. Yu , X. Wang , Z. Chen , Adv. Sci. 2021, 8, 2101051; 10.1002/advs.202101051 PMC8456284 34272930

[12] a) S.‐Q. Li , L. Zhang , T.‐T. Liu , Y.‐W. Zhang , C. Guo , Y. Wang , F.‐H. Du , Adv. Mater. 2022, 34, 2201801; 10.1002/adma.202201801 35417929

[13] Z. Li , H. Qin , W. Tian , L. Miao , K. Cao , Y. Si , H. Li , Q. Wang , L. Jiao , Adv. Funct. Mater. 2023, 34, 2301554.

[14] a) N. Yu , Y. Li , W. She , H. Li , H. Chen , W. Cheng , J. Chen , H. Liu , Y. Tu , Z. Huang , Y. Wan , L. Zou , X. Zhong , J. Luo , K. Guo , ACS Appl. Mater. Interfaces 2022, 14, 50827; 36326025 10.1021/acsami.2c13499

[15] a) J. Peng , W. Zhang , S. Wang , Y. Huang , J.‐Z. Wang , H.‐K. Liu , S.‐X. Dou , S.‐L. Chou , Adv. Funct. Mater. 2022, 32, 2111720;

[16] a) M. Z. Ge , C. Y. Cao , S. H. Li , Y. X. Tang , L. N. Wang , N. Qi , J. Y. Huang , K. Q. Zhang , S. S. Al‐Deyab , Y. K. Lai , Nanoscale 2016, 8, 5226; 26878901 10.1039/c5nr08341a

[17] H. Wang , Y. Wu , S. H. Liu , Y. Jiang , D. Shen , T. X. Kang , Z. Q. Tong , D. Wu , X. J. Li , C. S. Lee , Small Methods 2021, 5, 2001050. 10.1002/smtd.202001050 34927856

[18] S. Nanda , A. Gupta , A. Manthiram , Adv. Energy Mater. 2021, 11, 2000804.

[19] a) T. S. Wang , Y. C. Liu , Y. X. Lu , Y. S. Hu , L. Z. Fan , Energy Storage Mater. 2018, 15, 274;

[20] a) O. J. Dahunsi , S. Gao , J. Kaelin , B. Li , I. A. B. Razak , B. An , Y. Cheng , Nanoscale 2023, 15, 3255; 36723051 10.1039/d2nr06120a

[21] a) B. Ma , Y. Lee , P. Bai , Adv. Sci. 2021, 8, 2005006; 10.1002/advs.202005006 PMC8224441 34194939

[22] a) Z. Wang , Z. Huang , H. Wang , W. Li , B. Wang , J. Xu , T. Xu , J. Zang , D. Kong , X. Li , H. Y. Yang , Y. Wang , ACS Nano 2022, 16, 9105; 35666854 10.1021/acsnano.2c01186

[23] S. Ni , J. Liu , D. Chao , L. Mai , Adv. Energy Mater. 2019, 9, 1803324.

[24] H. Li , J. Lang , S. Lei , J. Chen , K. Wang , L. Liu , T. Zhang , W. Liu , X. Yan , Adv. Funct. Mater. 2018, 28, 1800757.

[25] a) X. Zhang , F. Hao , Y. Cao , Y. Xie , S. Yuan , X. Dong , Y. Xia , Adv. Funct. Mater. 2021, 31, 2009778;

[26] a) H. Yang , H. Wang , W. Li , B. Tian , T. Xu , D. Kong , S. Huang , K. Liu , X. Li , H. Y. Yang , Y. Wang , J. Mater. Chem. A 2022, 10, 16842;

[27] a) P. Liu , L. Miao , Z. Sun , X. Chen , Y. Si , Q. Wang , L. Jiao , Angew. Chem., Int. Ed. 2023, 62, e202312413; 10.1002/anie.202312413 37798812

[28] a) M. Zhu , G. Wang , X. Liu , B. Guo , G. Xu , Z. Huang , M. Wu , H.‐K. Liu , S.‐X. Dou , C. Wu , Angew. Chem., Int. Ed. 2020, 59, 6596; 10.1002/anie.201916716 31989734

[29] a) B. Sun , P. Li , J. Q. Zhang , D. Wang , P. Munroe , C. Y. Wang , P. H. L. Notten , G. X. Wang , Adv. Mater. 2018, 30, 1801334; 10.1002/adma.201801334 29855109

[30] a) X. Xu , C. Niu , M. Duan , X. Wang , L. Huang , J. Wang , L. Pu , W. Ren , C. Shi , J. Meng , B. Song , L. Mai , Nat. Commun. 2017, 8, 460; 28878210 10.1038/s41467-017-00211-5 PMC5587687

[31] a) J. Kim , J. Kim , J. Jeong , J. Park , C.‐Y. Park , S. Park , S. G. Lim , K. T. Lee , N.‐S. Choi , H. R. Byon , C. Jo , J. Lee , Energy Environ. Sci. 2022, 15, 4109;

[32] a) Y. J. Liu , M. Bai , D. Du , X. Y. Tang , H. L. Wang , M. Zhang , T. Zhao , F. Liu , Z. Q. Wang , Y. Ma , Energy Environ. Mater. 2023, 6, e12350;

