# Supplementary material for: Superior Sodium Metal Anodes Enabled by 3D Hierarchical Metallic Scaffolds with Enhanced Sodiophilicity
Source: Adv Sci (Weinh). 2025 Apr 18;12(25):2500756. doi: 10.1002/advs.202500756 (PMC12224975; doi:10.1002/advs.202500756)
Supplement: Supplementary file 1 — Supporting Information [file ADVS-12-2500756-s001.docx]

Supporting Information

Superior Sodium Metal Anodes Enabled by 3D Hierarchical Metallic Scaffolds with Enhanced Sodiophilicity

Chong Chen^1,a^, Rui Yang^1,a^, Jie Zhu^1^, Wenjiao Yao^1,2,3^*, and Yongbing Tang^1,3^*

Dr. C. Chen, Dr. R. Yang, J. Zhu, Dr. W. J. Yao, Prof. Y. B. Tang

Advanced Energy Storage Technology Research Center, Shenzhen Institutes of Advanced Technology, Chinese Academy of Sciences, Shenzhen 518055, China.

Dr. W. J. Yao, Prof. Y. B. Tang

Shenzhen College of Advanced Technology, University of Chinese Academy of Sciences, Shenzhen 518055, China

Dr. W. J. Yao

Shenzhen Key Laboratory of Energy Materials for Carbon Neutrality, Shenzhen 518055, China

^a^ These authors contribute equally to this work.

E-mail: wj.yao@siat.ac.cn, tangyb@siat.ac.cn

**Experimental Section**

*Synthesis of c-TiO_2_ NTAs.* All the reagents were of analytical grade and used as received without further purifications. Briefly, TiO_2_ nanotube arrays (TiO_2_ NTAs) was synthesized by typical electrochemical anodic oxidation process. First, Ti foils were ultrasonically cleaned in acetone, ethanol, and deionized water, respectively, for 30 min, and dried in an oven. The Ti foil was then immersed into an NH_4_F (0.12 M) electrolyte consisting of ethylene glycol (900 mL, Sinopharm Chemical Reagent Co., Ltd.) and deionized water (100 mL). Electrochemical anodization was carried out in a two electrode set-up with another clean Ti foil as counter electrode. The Ti foils were anodized in the electrolyte at an applied potential of 60 V for 6 h. After anodization, the sample was washed by ethanol and deionized water several times. Afterward, the TiO_2_ NTAs were annealed at 400°C in the air for 2 hours to obtain the annealed c-TiO_2_ NTAs products. The ramping rate was 2 °C min^-1^.

*Synthesis of Ag@TiO_2_ NTAs.* Ag@TiO_2_ NTAs were prepared by depositing silver nanocrystals on c-TiO_2_ NTAs precursors using an ultrasonication-assisted *in-situ* deposition method. First, 1.4 mL ammonia solution and 0.5 g AgNO_3_ were added into 40 mL deionized water. Then, polyvinylpyrrolidone (PVP-K30, 0.4 g) was added to the solution and mixed using an ultrasonic generator with a frequency of 37 kHz for 5 minutes. Subsequently, c-TiO_2_ NTAs were immersed into the solution, followed by the addition of 0.9 mg glucose. After ultrasonication for another 20 minutes at 37 kHz, the Ag@TiO_2_ NTAs samples were obtained.

**Materials characterization**

The morphology of the samples was investigated by field-emission scanning electron microscope (FESEM; JEOL JSM7800F)) and transmission electron microscope (TEM; JEOL, HT7700) equipped with energy-dispersive X-ray (EDX) spectroscopy. High-resolution TEM (HRTEM) images, high-angle annular dark-field scanning TEM (HAADF-STEM) images and corresponding element mappings were collected on a high-resolution field emission TEM (JEOL, JEM-2100F) with EDX and STEM attachments. The crystal phase was analyzed by X-ray diffraction (XRD) on a Bruker D2 Phaser X-ray diffractometer with Ni filtered Cu K*α* radiation (*λ* = 1.5406 Å) at a voltage of 30 kV and a current of 10 mA. The surface chemical states of the samples were determined by an ESCALAB 250 X-ray photoelectron spectroscopy (XPS) system with an Al K*α* radiation source. The electrolyte wettability of the samples were estimated by a contact angle measuring device (CA-100C, ShangHai Innuo, China). In addition, the in-situ optical visualization was performed at a polarizing optical microscope (BX53, Olympus, Japan) by simultaneous electrodeposition of Na on the surface of electrodes.

**Electrochemical measurements.**

All of the batteries (CR2032 coin-type cells) were assembled in an argon-filled glovebox (moisture and oxygen < 0.1 ppm) and tested at 25 °C. Before electrochemical tests, all assembled batteries were rested for 10 h. In half-cells, Ag@TiO_2_ NTAs, c-TiO_2_ NTAs, and Ti foils were used as the working electrode (12 mm) and bare Na foil as the reference and counter electrode (16 mm), Whatman glass fiber as the separator and 1.0 M NaClO_4_ in Ethylene carbonate (EC)/Dimethyl carbonate (DEC) = 1:1 volume ratio with 5% FEC as the organic electrolyte. In the symmetric cell, 6 mAh cm^-2^ of Na was pre-deposited onto the working electrodes (Ti foils, c-TiO_2_ NTAs, and Ag@TiO_2_ NTAs). The electrolyte solution was 1 M sodium hexafluorophosphate (NaPF_6_) in diglyme. Before galvanostatic Na plating/stripping cycles, all of the symmetric cells need to be activated preferentially at 0.5 mA cm^-2^ with an areal capacity of 0.5 mAh cm^-2^ for 5 cycles. In the full cell test, the cathode was Na_3_V_2_(PO_4_)_3_ (NVP), and the electrolyte was the same as that of the symmetric cell. The NVP, PVDF and Super P were mixed with a weight ratio of 8:1:1 into N-methyl- 2-pyrrolidone (NMP) solvent. The homogeneous slurry was cast onto the Al foil and dried in the vacuum oven overnight at 80°C. The areal mass loading for the NVP cathodes was about 2 mg cm^-2^. All electrodes were cut into round disks with a diameter of 12 mm. The Ag@TiO_2_-Na||NVP full cells were tested by pairing pre-deposited Ag@TiO_2_ NTAs anode (6 mAh cm^-2^) with the NVP cathode. EIS was conducted in the range of 0.01 to 10^6^ Hz with an amplitude of 10 mV. The electrochemical performances of the cells are evaluated on a Neware battery test system.

**Computational Details.**

The structures, energies and electron structures of c-TiO_2_, fully sodiated c-TiO_2_, and Ag were optimized through the density functional theory (DFT),^[1,2]^ performed with the Vienna Ab initio simulation package.^[3]^ The generalized gradient approximation with Perde-Burke-Ernzerhof function were applied for the exchange-correlation interaction.^[4]^ The cutoff energy of projector-augmented wave potential was set to be 450 eV for structural relaxation.^[5]^ The electronic relaxation loops terminated when the energy difference between two steps is lower than 1.0×10^-5^ eV. Before building the surface, the structures of c-TiO_2_, fully sodiated c-TiO_2_, and Ag were firstly optimized in crystalline form. And the density of electronic states (DOS) of each material was obtained by the crystal structure. To simulate the surface structures of c-TiO_2_, fully sodiated c-TiO_2_, and Ag, a vacuum layer of about 30 Å were applied, and the cell structures were fixed during subsequential relaxations. Meanwhile, the bottom two layers of atoms were fixed to keep the base properties close to the crystal. The adsorption energy (*E*_ad_) between fully sodiated c-TiO_2_/Ag surface and Na atom is defined as following:

$$E_{\mathrm{ad}}=E_{\mathrm{Complex}}-E_{\mathrm{surface}}-E_{\mathrm{Na}}$$

where *E*_Complex_ is the total energy of the Na atom adsorbed on the surface of fully sodiated c-TiO_2_/Ag, $E_{\mathrm{surface}}$ is the energy of fully sodiated c-TiO_2_/Ag surface, and $E_{\mathrm{Na}}$ is the energy of Na atom.


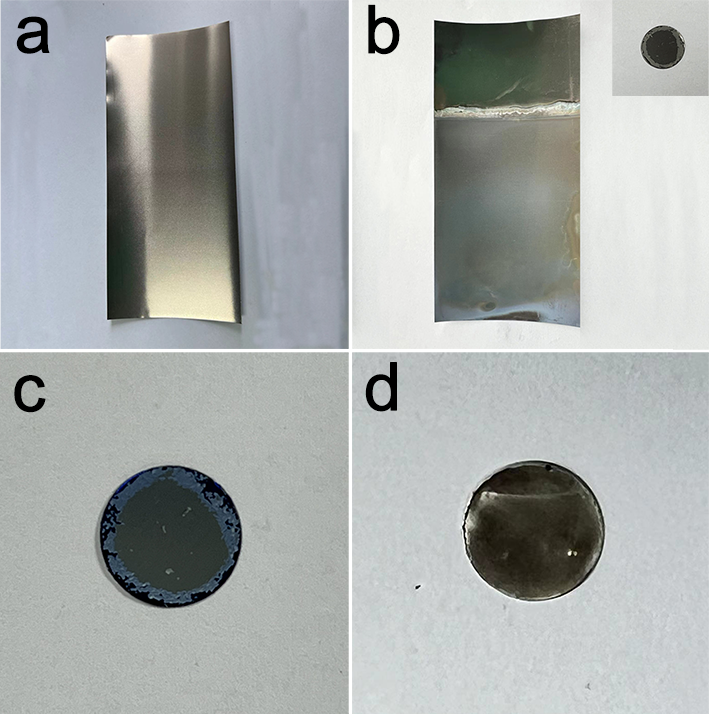


**Figure S1**. Digital photos of (a) Ti Foil, (b) TiO_2_ NTAs, (c) c-TiO_2_ NTAs and (d) Ag@TiO_2_ NTAs.

**
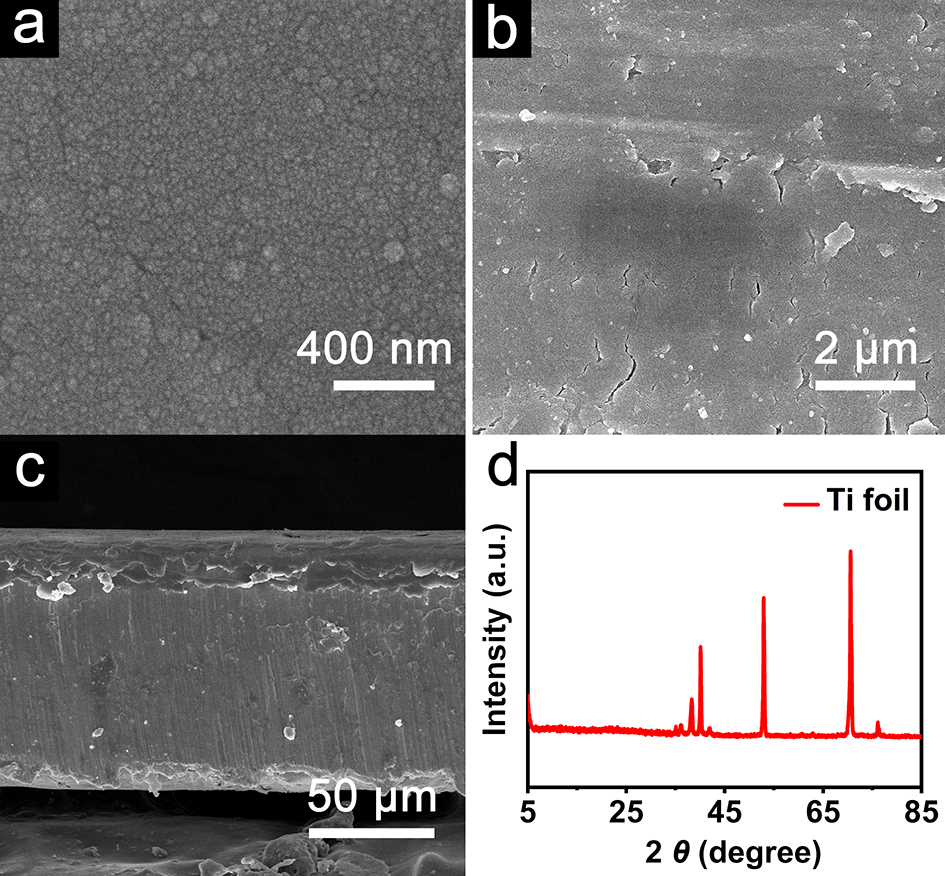
**

**Figure S2**. (a,b) FESEM images, (c) cross-sectional FESEM image and (d) XRD pattern of Ti foil.


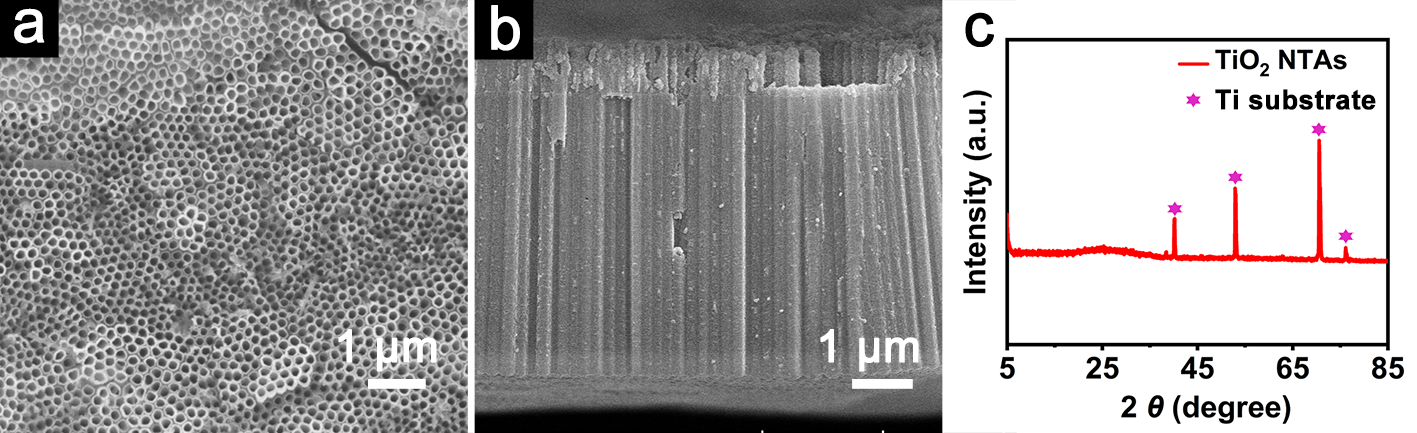


**Figure S3**. (a) FESEM image, (b) cross-sectional FESEM image and (c) XRD pattern of TiO_2_ NTAs.


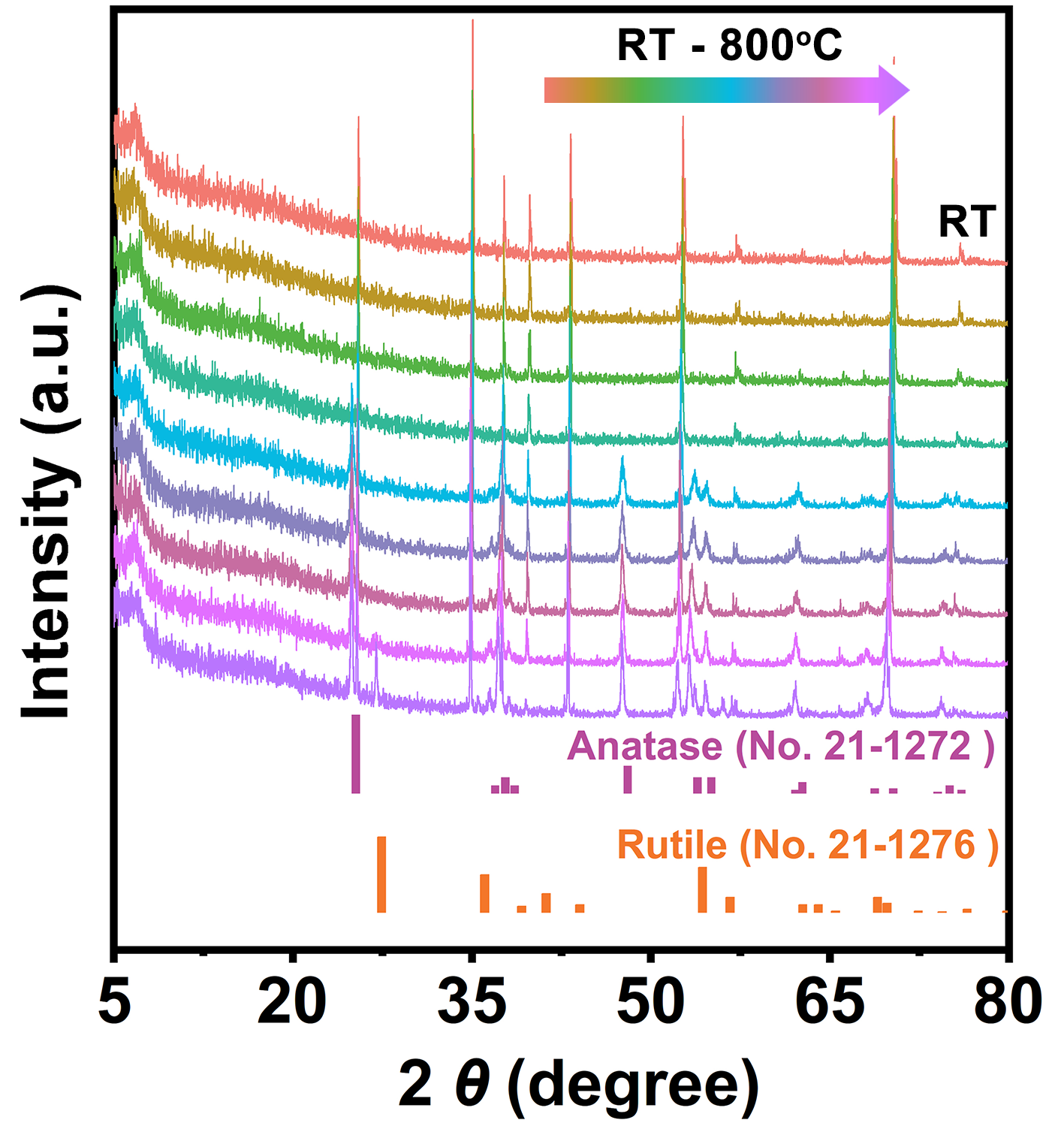


**Figure S4**. *In-situ* XRD patterns of TiO_2_ NTAs to reveal the corresponding structural and phase evolutions during heating from RT to 800 ℃.


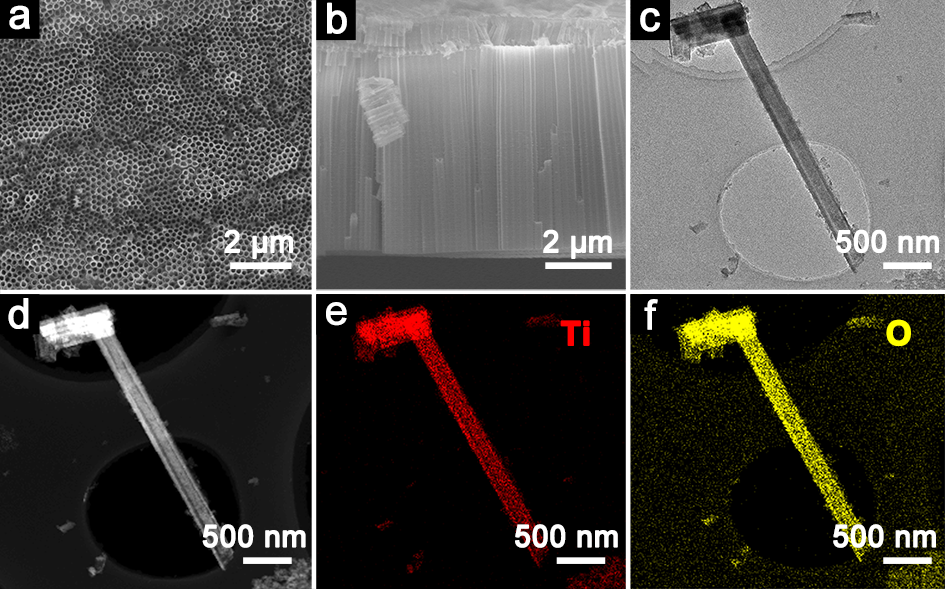


**Figure S5**. (a) FESEM image and (b) the cross-sectional image of c-TiO_2_ NTAs. (c) TEM, (d) HAADF-STEM and (e,f) the corresponding elemental mapping images of a single c-TiO_2_ nanotube.


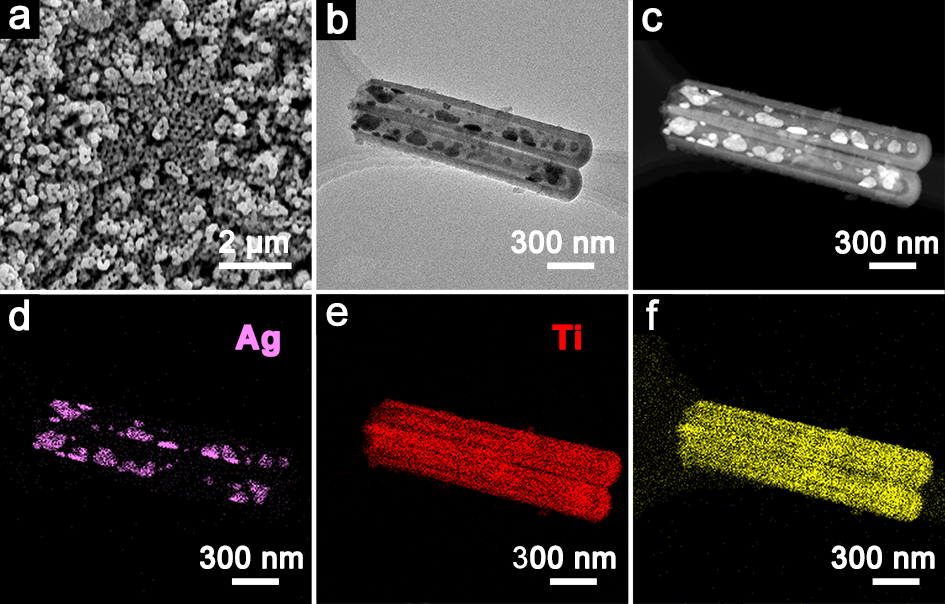


**Figure S6**. (a) FESEM image, (b) TEM, (c) HAADF-STEM and (d-f) the corresponding elemental mapping images of Ag@TiO_2_ NTAs.


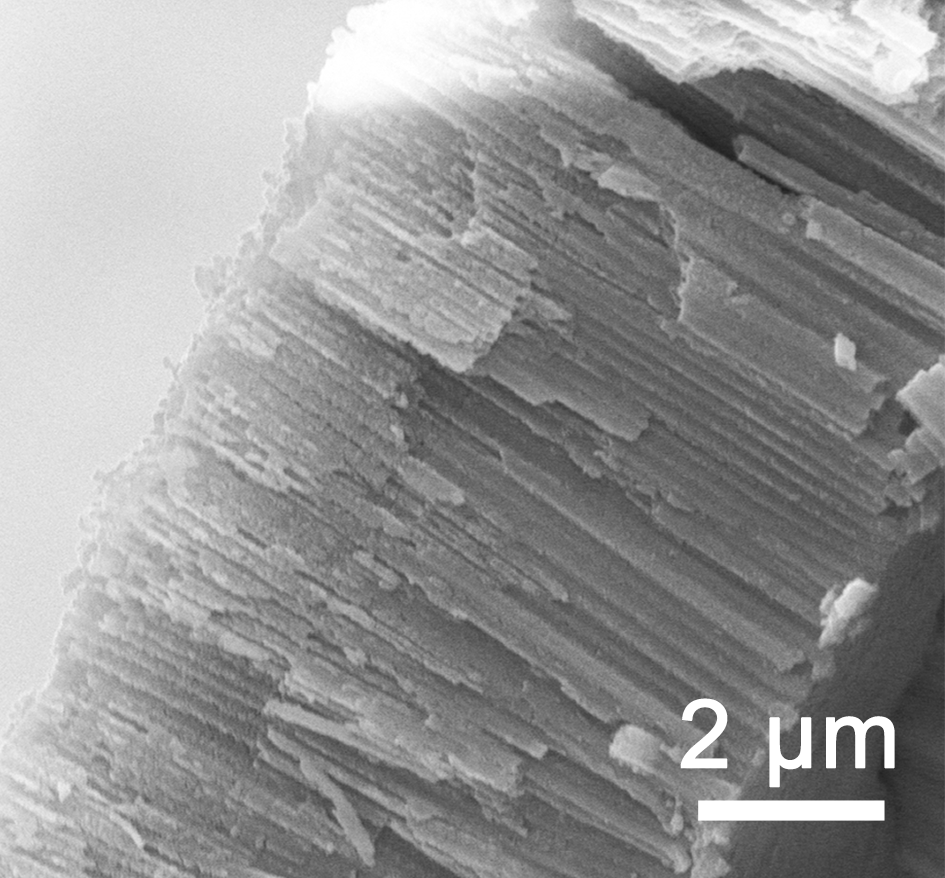


**Figure S7**. The cross-sectional FESEM image of Ag@TiO_2_ NTAs.


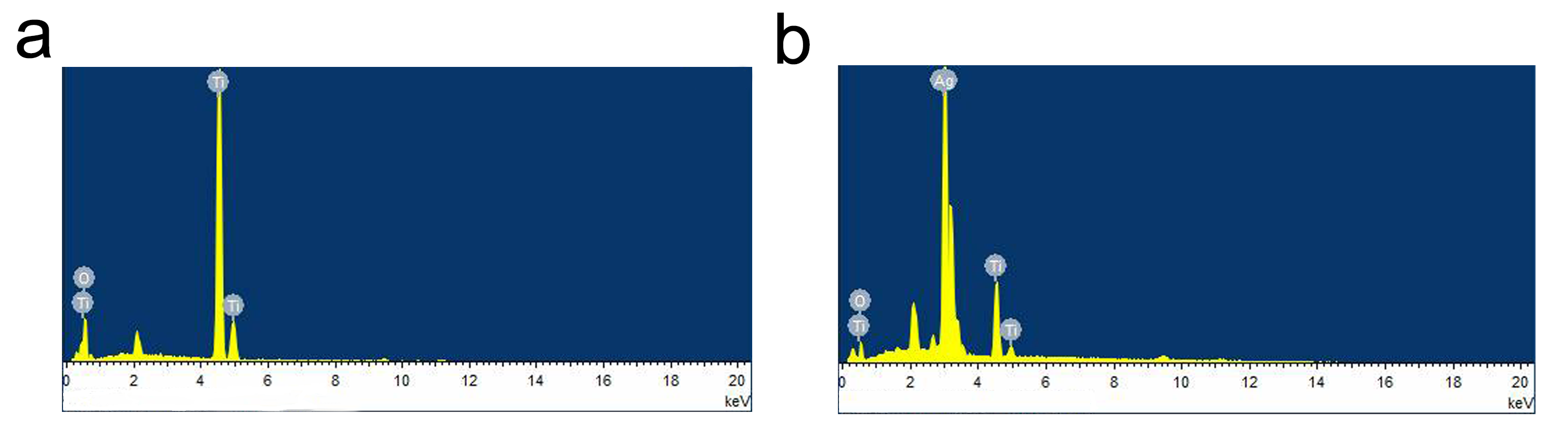


**Figure S8**. EDX results of (a) c-TiO_2_ NTA and (b) Ag@TiO_2_ NTA.


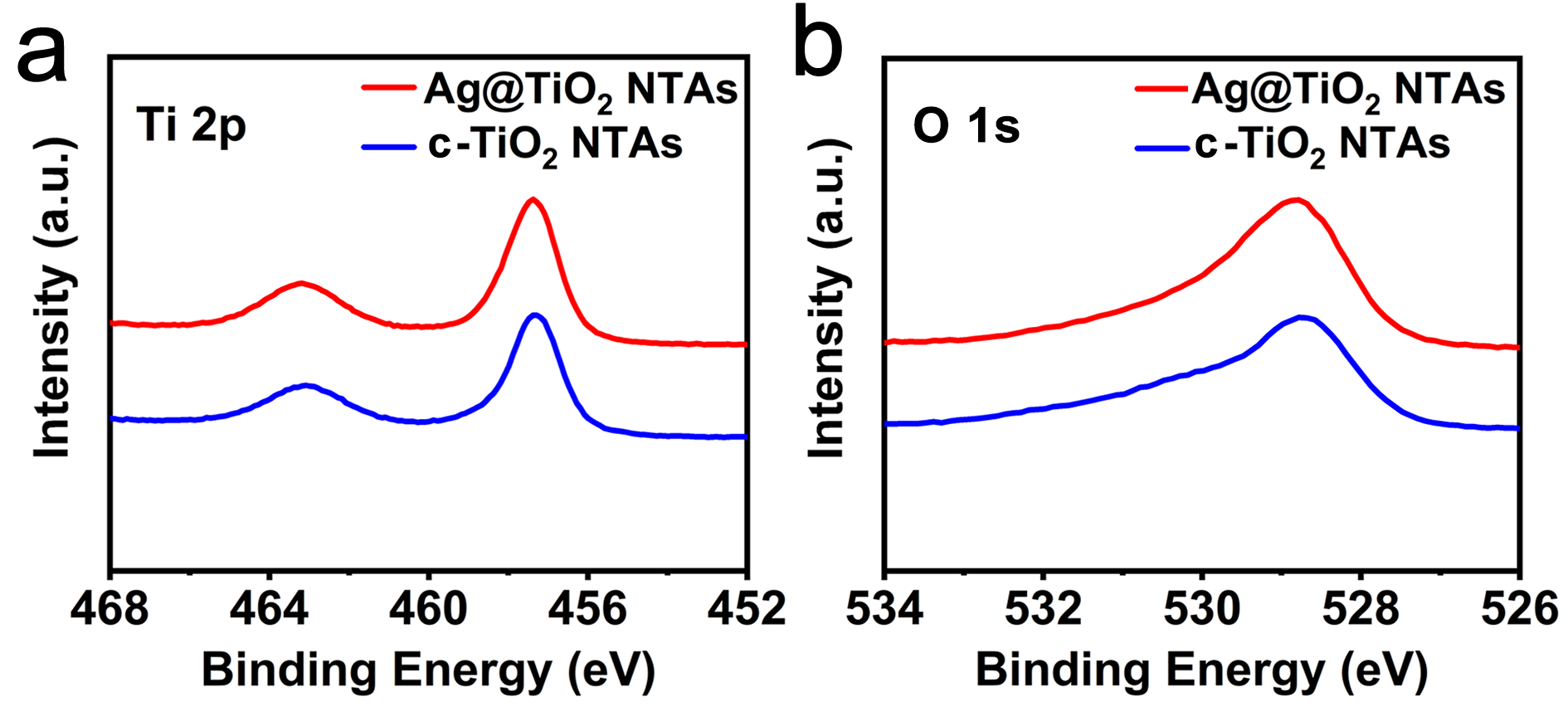


**Figure S9**. High-resolution XPS spectra of (a) Ti 2p and (b) O 1s for the c-TiO_2_ NTAs and Ag@TiO_2_ NTAs.


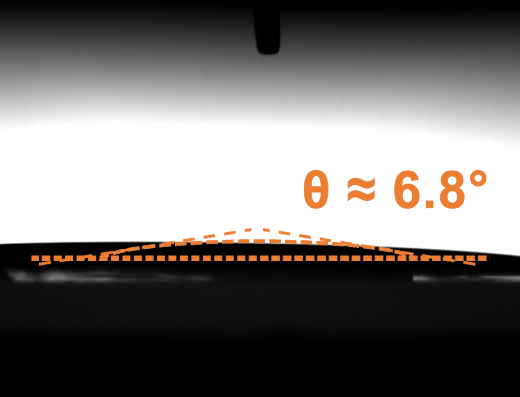


**Figure S10**. Contact angle tests of diglyme electrolyte on c-TiO_2_ NTAs.


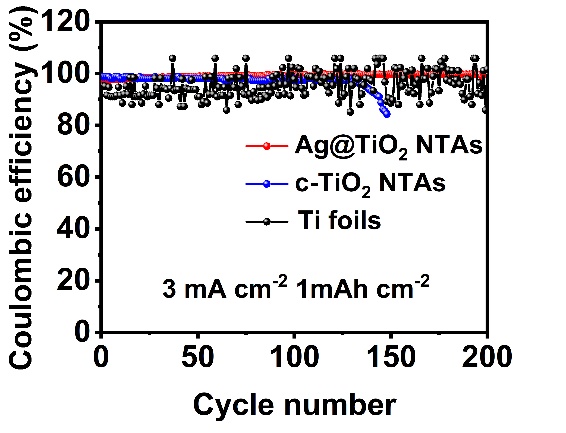


**Figure S11**. Coulombic efficiency of Na plating/stripping on the different hosts with a plating capacity of 1 mAh cm^-2^ at 3 mA cm^-2^.


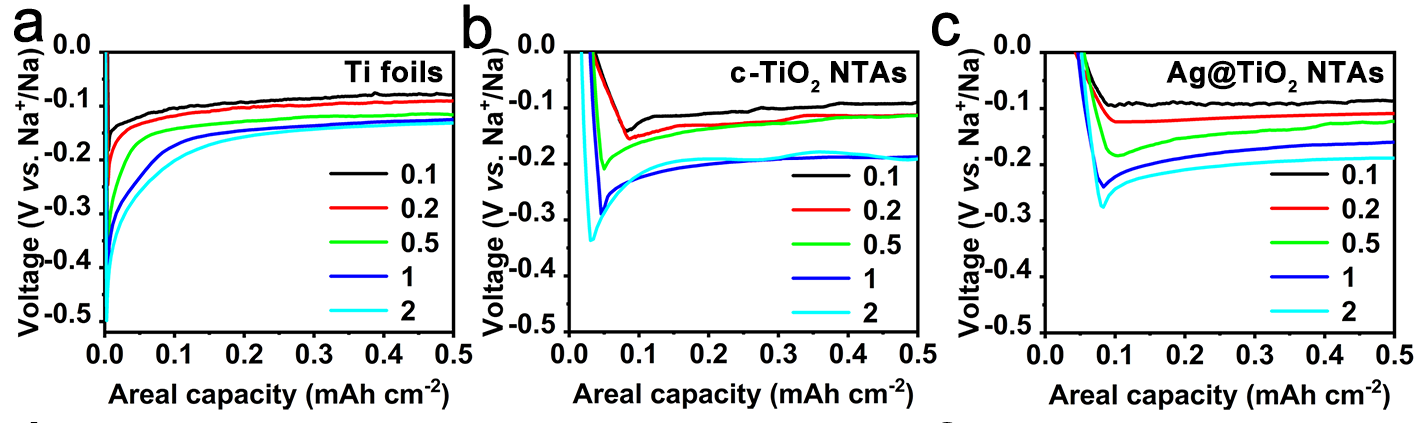


**Figure S12**. Nucleation potentials of Na metal plating on the (a) Ti foils, (b) c-TiO_2_ NTAs and (c) Ag@TiO_2_ NTAs hosts at various current densities.


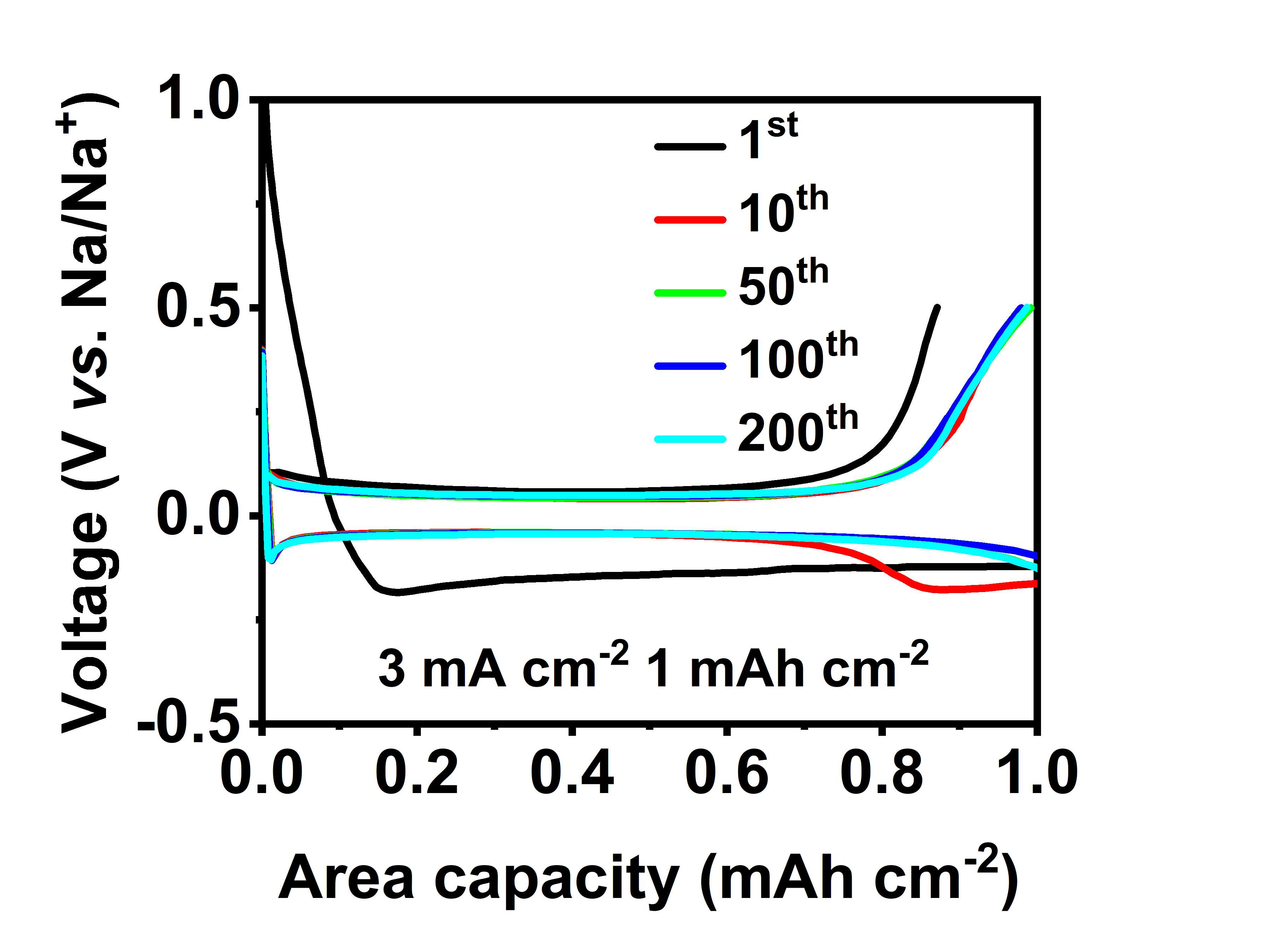


**Figure S13**. Voltage-capacity profiles of the 1^st^, 10^th^, 50^th^, 100^th^, and 200^th^ cycle of Ag@TiO_2_ NTAs electrode at a current density of 3 mA cm^-2^ and a deposition capacity of 1 mAh cm^-2^.


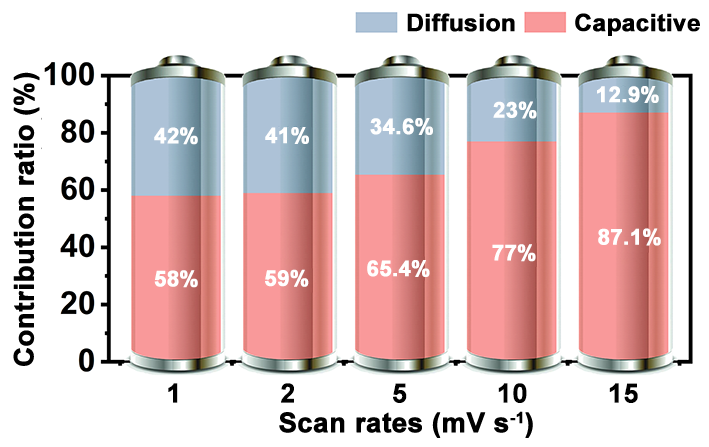


**Figure S14**. The capacitive contribution ratios to the total capacity of Ag@TiO_2_ NTAs.


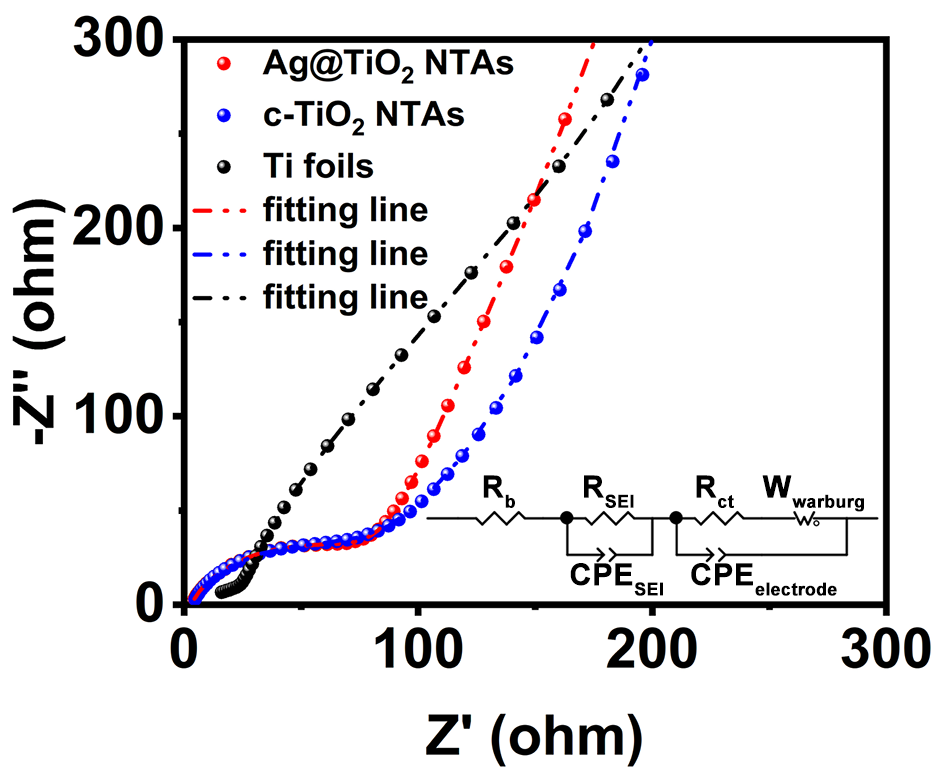


**Figure S15**. The equivalent circuit model to fit the Nyquist plots of Ag@TiO_2_ NTAs, c-TiO_2_ NTAs and Ti foils, where *R*_b_, *R*_SEI_ and *R*_ct_ represent the resistance of the bulk electrolyte, the Na^+^ diffusion through SEI and charge transfer resistance, respectively.


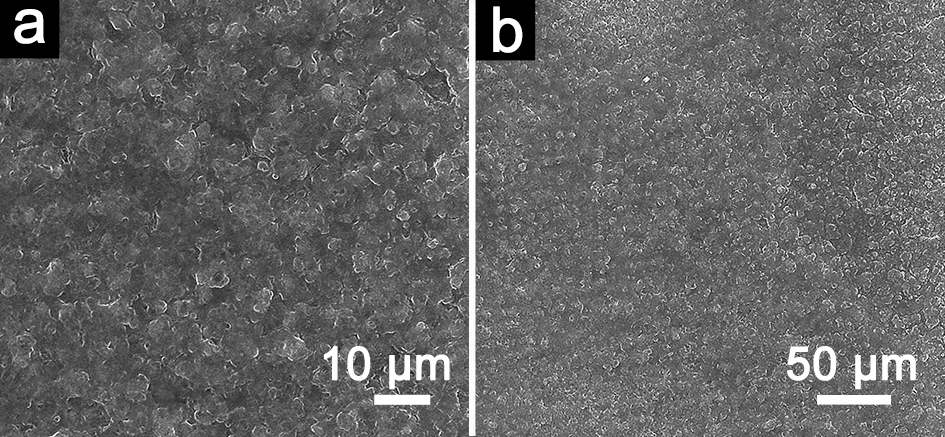


**Figure S16**. FEESM images of the Ag@TiO_2_ NTAs hosts after Na plating with a capacity 20 mAh cm^-2^.


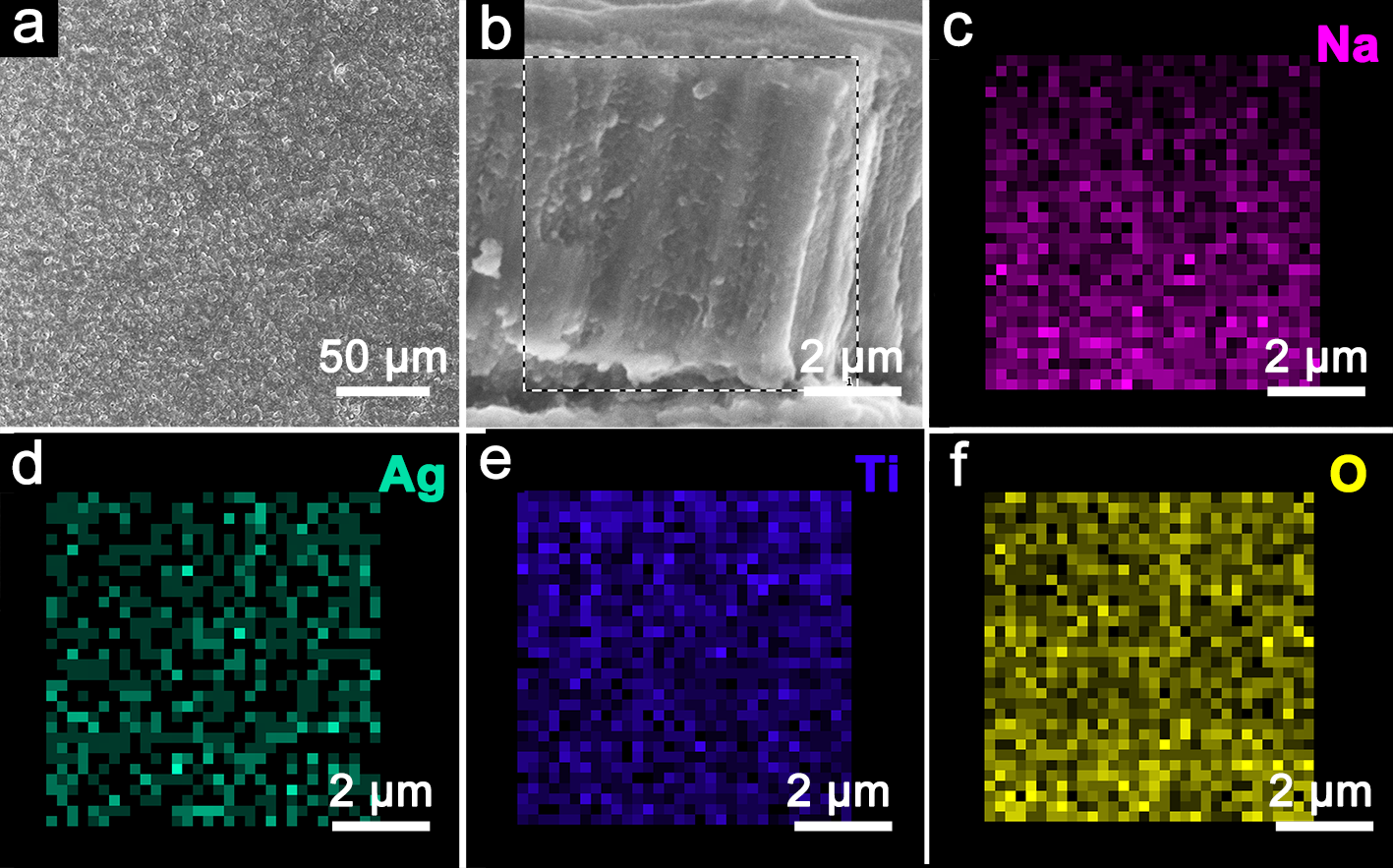


**Figure S17**. (a) Top-view, (b) cross-sectional FESEM image and (c-f) the corresponding elemental mapping images of Ag@TiO_2_-Na electrode at the plating capacity of 6 mAh cm^-2^.


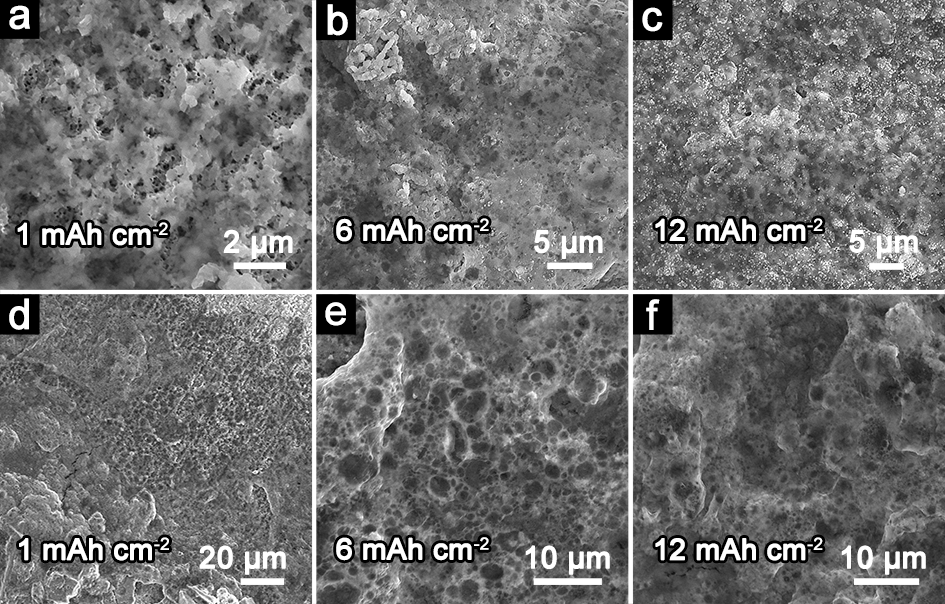


**Figure S18**. FEESM images of (a-c) the c-TiO_2_ NTAs hosts and (d-f) Ti foils after Na plating with a capacity of (a,d) 1 mAh cm^-2^, (b,e) 6 mAh cm^-2^ and (c,f) 12 mAh cm^-2^.


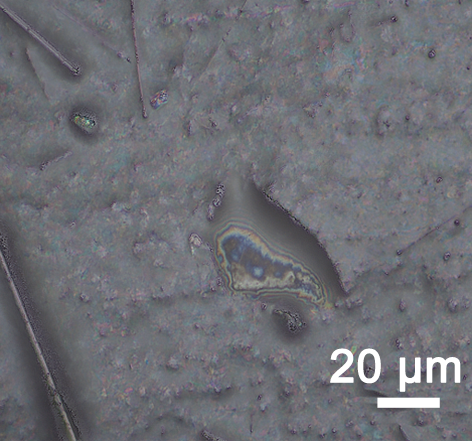


**Figure S19**. Surface FESEM images of c-TiO_2_ NTAs electrodes in symmetric cell after 100 cycles at 1 mA cm^-2^.


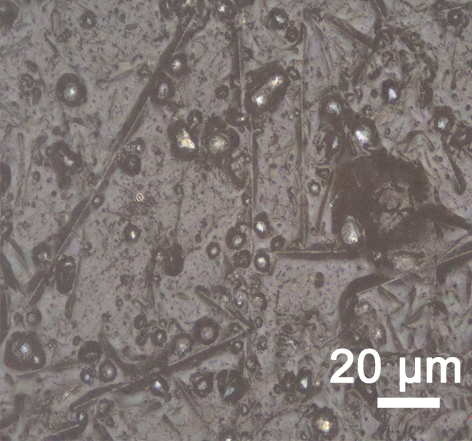


**Figure S20**. Surface FESEM images of Ti foils electrodes in symmetric cell after 100 cycles at 1 mA cm^-2^.


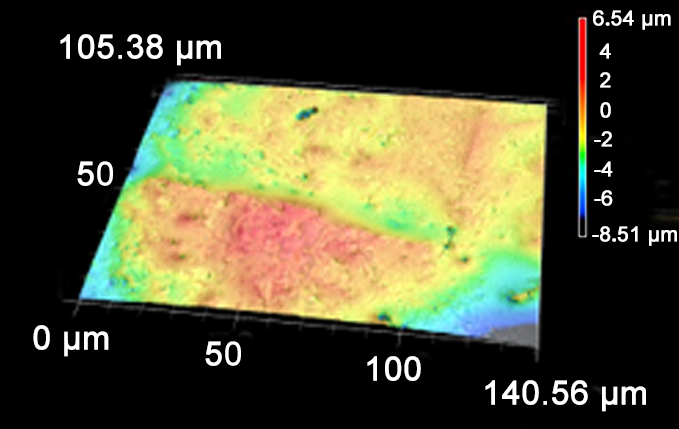


**Figure S21**. 3D LSCM images of c-TiO_2_ NTAs after 100 cycles at 1 mA cm^–2^.


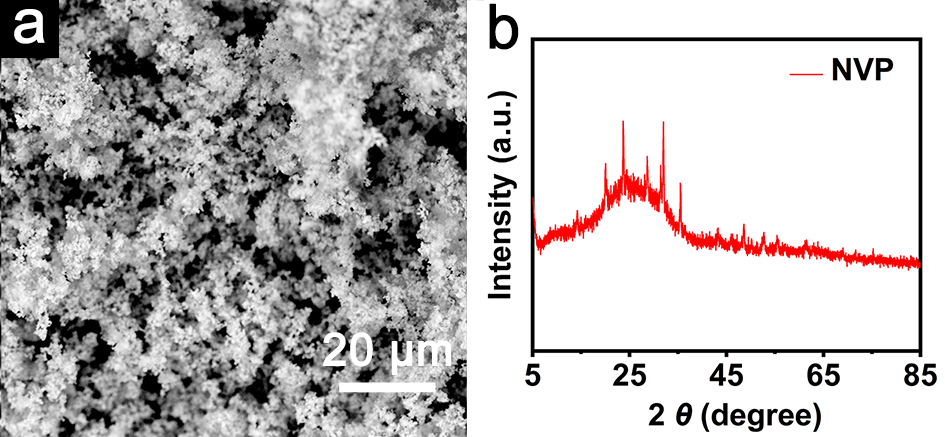


**Figure S22**. (a) FESEM image and (b) XRD pattern of NVP.


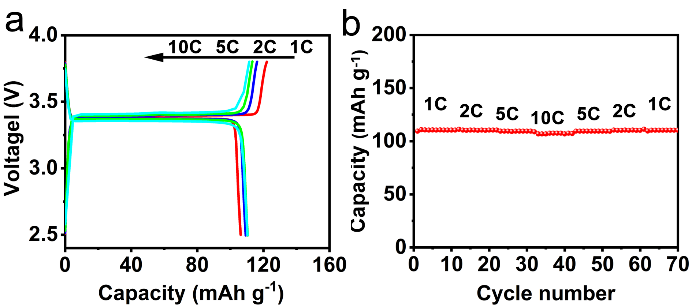


**Figure S23**. (a) Discharge and charge voltage profiles of the NVP||Na cell at different rates. (b) Rate performances of the NVP||Na cell.


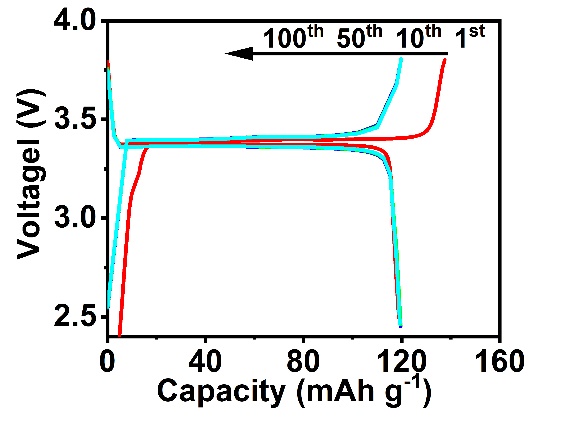


**Figure S24**. Discharge and charge voltage profiles of the NVP||Na cell at the 1^st^, 10^th^, 50^th^, and 100^th^ cycles at 1 C.


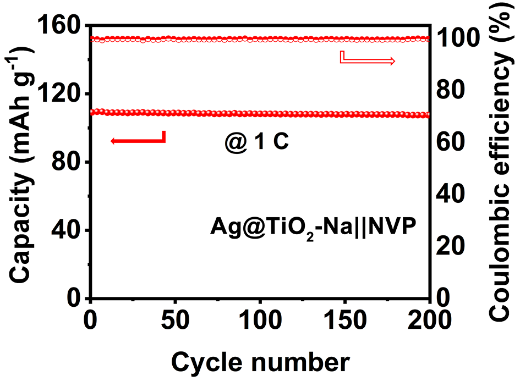


**Figure S25**. Cycling stability of Ag@TiO_2_-Na||NVP full cell at 1 C.


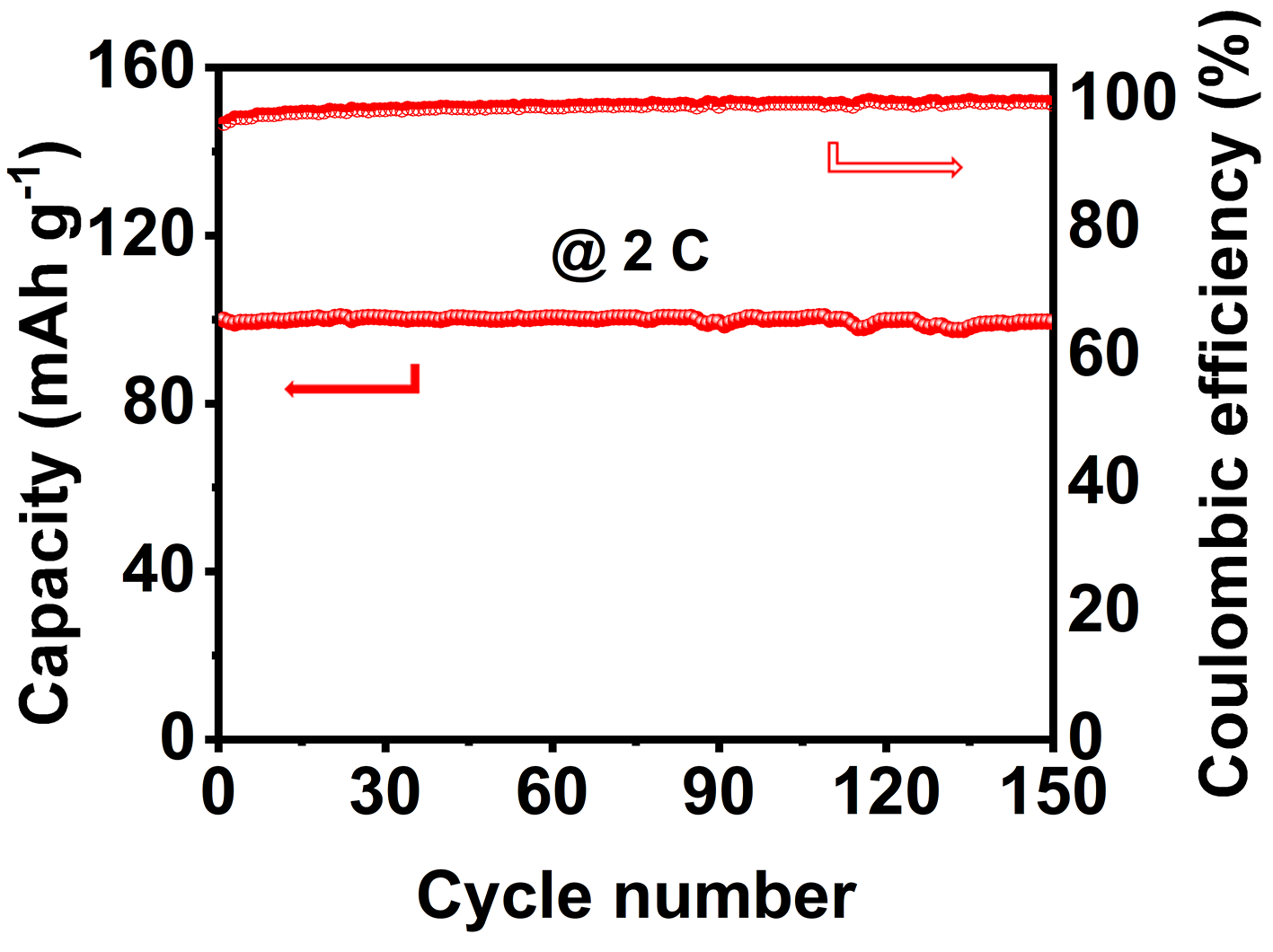


**Figure S26**. Cycling performance of the full cells with a low N/P ratio ≈4.5 at 2 C.


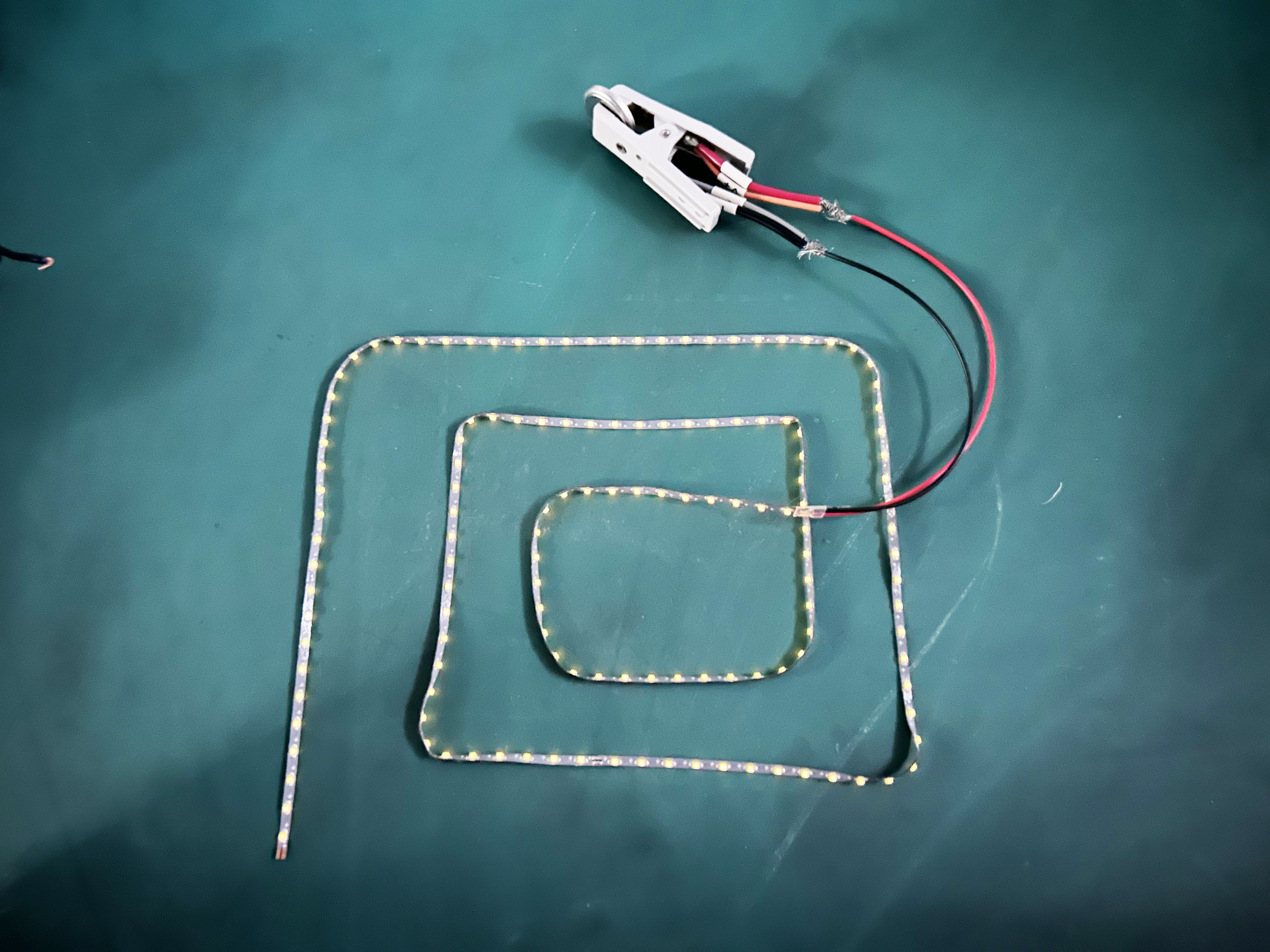


**Figure S27**. The optical photograph showing that LED arrays are lightened by Ag@TiO_2_-Na||NVP device.

**Table S1**. Resistance values acquired by modeling the experimental impedance of the Ag@TiO_2_ NTAs, c-TiO_2_ NTAs and Ti foils.

|  | *R*_SEI_ (Ω) | Error (%) | *R*_ct_ (Ω) | Error (%) |
| --- | --- | --- | --- | --- |
| Ag@TiO_2_ NTAs | 3.244 | 3.4954 | 92.77 | 4.4836 |
| c-TiO_2_ NTAs | 3.839 | 2.7554 | 96.79 | 3.5906 |
| Ti foils | 4.83 | 13.397 | 27.01 | 19.722 |

**Table S2**. Comparison of recently reported hosts of the symmetric cell performance with previously reported works.

| Host | Current density  (mA cm^-2^) | | Capacity  (mAh cm^-2^) | Lifespan | Ref. |
| --- | --- | --- | --- | --- | --- |
| Ag@TiO_2_ NTAs | 1 | 1 | | 3600 h | This work |
|  | 4 | 4 | | 2000 h |  |
| MgF_2_@RGO | 0.5 | 0.5 | | 1600 h | [6] |
|  | 1 | 1 | | 500 h |  |
|  | 2 | 2 | | 300 h |  |
| CoSA@NC | 1 | 1 | | 2200 h | [7] |
| CNF@Ag | 2 | 1 | | 500 h | [8] |
|  | 3 | 6 | | 1000 h |  |
| Ti_3_C_2_T*_x_*/CNT NAFs | 3 | 3 | | 1500 h | [9] |
|  | 5 | 10 | | 1500 h |  |
| N-CSs | 2 | 2 | | 1500 h | [10] |
| Ti_3_C_2_ MXene@g-C_3_N_4_ | 1 | 1 | | 700 h | [11] |
|  | 3 | 3 | | 100 cycles |  |
| Ti_3_C_2_T*_x_*-CC | 3 | 1 | | 1000 h | [12] |
|  | 5 | 1 | | 300 h |  |
| Sn@LIG@Cu | 1 | 1 | | 2400 h | [13] |
|  | 10 | 10 | | 1000 h |  |
| 3D flexible carbon felt | 1 | 2 | | 480 h | [14] |
|  | 3 | 2 | | 160 h |  |
| Bi⊂CNs | 1 | 2 | | 2800 h | [15] |
|  | 2 | 2 | | 1150 h |  |
| NSCA-31 | 1 | 1 | | 700 h | [16] |
|  | 2 | 1 | | 550 h |  |
| STNTs-Ag | 1 | 0.5 | | 1400 h | [17] |
|  | 1 | 1 | | 700 h |  |
| C@Sb | 1 | 1 | | 2400 h | [18] |
|  | 1 | 2 | | 1188 h |  |
| a-CNTs | 1 | 1 | | 250 h | [19] |
|  | 3 | 1 | | 1100 h |  |

**Table S3**. Comparison of reported NVP cells with modified Na anodes with previously reported works.

| Anode Materials | Current density (C) | Capacity  (mAh g^-1^) | Cycle numbers | Ref. |
| --- | --- | --- | --- | --- |
| Ag@TiO_2_ NTAs | 8 | 82 | 2000 | This work |
| (Ti_3_C_2_T*_x_*)-coated  carbon cloth@infused Na | 4.2 | 88 | 300 | [12] |
| Na-Na_2_S-carbon hybrid | 1 | 54 | 500 | [20] |
| Activated SnS-graphene  membrane@Na | 0.4 | 74 | 400 | [21] |
| N-doped hollow carbon fibers@deposited Na | - | 94 | 200 | [22] |
| Porous carbon  nanofibers@deposited Na | 1 | 75 | 900 | [23] |
| 3D printed  rGO/CNT@deposited Na | 0.85 | 68 | 100 | [24] |
| Na | 1 | 93 | 400 | [25] |
| Co-graphene/carbon cloth@infused Na | 1.7 | 65 | 370 | [26] |
| Zn,Mg-doped NASICON/Na | 0.2 | 80 | 300 | [27] |
| FCTF/Na | 2 | 97 | 700 | [28] |

References

[1] P. Hohenberg, W. Kohn, *Phys. Rev.* **1964**, 136, B864.

[2] W. Kohn, L. J. Sham, *Phys. Rev.* **1965**, 140, A1133.

[3] G. Kresse, J. Hafner, *Phys. Rev. B* **1993**, 47, 558.

[4] J. P. Perdew, K. Burke, M. Ernzerhof, *Phys. Rev. Lett.* **1997**, 78, 1396.

[5] P. E. Blöchl, *Phys. Rev. B* **1994**, 50, 17953.

[6] L. Zhao, Z. Hu, Z. Huang, Y. Tao, W.-H. Lai, A. Zhao, Q. Liu, J. Peng, Y. Lei, Y.-X. Wang, Y. Cao, C. Wu, S.-L. Chou, H. K. Liu, S. X. Dou, *Adv. Energy Mater.* **2022**, *12*, 2200990.

[7] Y. J. Li, P. Xu, J. R. Mou, S. F. Xue, S. M. Huang, J. H. Hu, Q. F. Dong, C. H. Yang, M. L. Liu, *Small Methods* **2021**, *5*, 2100833.

[8] L. L. Mo, A. L. Chen, Y. Ouyang, W. Zong, Y. E. Miao, T. X. Liu, *ACS Appl. Mater. Interfaces* **2021**, *13*, 48634.

[9] S. Kandula, E. Kim, C. W. Ahn, J. Lee, B. Yeom, S. W. Lee, J. Cho, H.-K. Lim, Y. Lee, J. G. Son, *Energy Storage Mater.* **2023**, *63*, 103024.

[10] B. Huang, S. Sun, J. Wan, W. Zhang, S. Liu, J. Zhang, F. Yan, Y. Liu, J. Xu, F. Cheng, Y. Xu, Y. Lin, C. Fang, J. Han, Y. Huang, *Adv. Sci.* **2023**, *10*, 2206845.

[11] C. Bao, J. Wang, B. Wang, J. Sun, L. He, Z. Pan, Y. Jiang, D. Wang, X. Liu, S. X. Dou, J. Wang, *ACS Nano* **2022**, *16*, 17197.

[12] Y. Fang, R. Lian, H. Li, Y. Zhang, Z. Gong, K. Zhu, K. Ye, J. Yan, G. Wang, Y. Gao, Y. Wei, D. Cao, *ACS Nano* **2020**, *14*, 8744.

[13] H. Xiao, Y. Li, W. Chen, T. Xie, H. Zhu, W. Zheng, J. He, S. Huang, *Small* **2023**, *19*, 2303959.

[14] S.-S. Chi, X.-G. Qi, Y.-S. Hu, L.-Z. Fan, *Adv. Energy Mater.* **2018**, *8*, 1702764.

[15] L. Zhang, X. Zhu, G. Wang, G. Xu, M. Wu, H.-K. Liu, S.-X. Dou, C. Wu, *Small* **2021**, *17*, 2007578.

[16] X. Y. Zheng, W. J. Yang, Z. Q. Wang, L. Q. Huang, S. Geng, J. Y. Wen, W. Luo, Y. H. Huang, *Nano Energy* **2020**, *69*, 104387.

[17] J. S. Wang, R. Q. Lian, S. Zhao, L. T. Zheng, Y. Y. Huang, M. D. Wei, S. Mathur, Z. S. Hong, *Chem. Eng. J.* **2022**, *431*, 134272.

[18] G. Y. Wang, Y. Zhang, B. K. Guo, L. Tang, G. Xu, Y. J. Zhang, M. H. Wu, H. K. Liu, S. X. Dou, C. Wu, *Nano Lett.* **2020**, *20*, 4464.

[19] Y. Zhao, X. F. Yang, L. Y. Kuo, P. Kaghazchi, Q. Sun, J. N. Liang, B. Q. Wang, A. Lushington, R. Y. Li, H. M. Zhang, X. L. Sun, *Small* **2018**, *14*, 1703717.

[20] W. Wu, S. Hou, C. Zhang, L. Zhang, *ACS Appl. Mater. Interfaces* **2020**, *12*, 27300.

[21] W. Liu, Z. D. Chen, Z. Zhang, P. X. Jiang, Y. G. Chen, E. Paek, Y. X. Wang, D. Mitlin, *Energy Environ. Sci.* **2021**, *14*, 382.

[22] X. Y. Zheng, P. Li, Z. Cao, W. Luo, F. Z. Sun, Z. Q. Wang, B. Ding, G. X. Wang, Y. H. Huang, *Small* **2019**, *15*, 1902688.

[23] N. Mubarak, M. Ihsan-Ul-Haq, H. Huang, J. Cui, S. S. Yao, A. Susca, J. X. Wu, M. Y. Wang, X. H. Zhang, B. L. Huang, J. K. Kim, *J. Mater. Chem. A* **2020**, *8*, 10269.

[24] J. Yan, G. Zhi, D. Z. Kong, H. Wang, T. T. Xu, J. H. Zang, W. X. Shen, J. M. Xu, Y. M. Shi, S. G. Dai, X. J. Li, Y. Wang, *J. Mater. Chem. A* **2020**, *8*, 19843.

[25] X. Y. Liu, X. Y. Zheng, Y. M. Dai, W. Y. Wu, Y. Y. Huang, H. Y. Fu, Y. H. Huang, W. Luo, *Adv. Funct. Mater.* **2021**, *31*, 2103522.

[26] C. Lu, Z. X. Gao, B. Z. Liu, Z. X. Shi, Y. Y. Yi, W. Zhao, W. Y. Guo, Z. F. Liu, J. Y. Sun, *Adv. Funct. Mater.* **2021**, *31*, 2101233.

[27] P. W. Jaschin, C. R. Tang, E. D. Wachsman, *Energy Environ. Sci.* **2024**, *17*, 727.

[28] R. Zhuang, X. Zhang, C. Qu, X. Xu, J. Yang, Q. Ye, Z. Liu, S. Kaskel, F. Xu, H. Wang, *Sci. Adv.* **2023**, *9*, eadh8060.
